# Gamma-diversity partitioning of gobiid fishes (Teleostei: Gobiidae) ensemble along of Eastern Tropical Pacific: Biological inventory, latitudinal variation and species turnover

**DOI:** 10.1371/journal.pone.0202863

**Published:** 2018-08-31

**Authors:** Omar Valencia-Méndez, Fabián Alejandro Rodríguez-Zaragoza, Luis Eduardo Calderon-Aguilera, Omar Domínguez-Domínguez, Andrés López-Pérez

**Affiliations:** 1 Doctorado en Ciencias Biológicas y de la Salud, Universidad Autónoma Metropolitana-Iztapalapa, Ciudad de México, México; 2 Departamento de Ecología, CUCBA, Universidad de Guadalajara, Jalisco, México; 3 Departamento de Ecología Marina, Centro de Investigación Científica y de Educación Superior de Ensenada (CICESE), Ensenada, Baja California, México; 4 Facultad de Biología, Universidad Michoacana de San Nicolás de Hidalgo, Morelia, Michoacán, México; 5 Departamento de Hidrobiología, Universidad Autónoma Metropolitana-Iztapalapa, Ciudad de México, México; Department of Agriculture and Water Resources, AUSTRALIA

## Abstract

Gobies are the most diverse marine fish family. Here, we analysed the gamma-diversity (γ-diversity) partitioning of gobiid fishes to evaluate the additive and multiplicative components of α and β-diversity, species replacement and species loss and gain, at four spatial scales: sample units, ecoregions, provinces and realms. The richness of gobies from the realm Eastern Tropical Pacific (ETP) is represented by 87 species. Along latitudinal and longitudinal gradients, we found that the γ-diversity is explained by the β-diversity at both spatial scales, ecoregions and provinces. At the ecoregion scale, species are diverse in the north (Cortezian ecoregion) and south (Panama Bight ecoregion) and between insular and coastal ecoregions. At the province scale, we found that the species turnover between the warm temperate Northeast Pacific (WTNP), Tropical East Pacific (TEaP) and the Galapagos Islands (Gala) was high, and the species nestedness was low. At the ecoregion scale, historical factors, and phylogenetic factors have influenced the hotspots of gobiid fish biodiversity, particularly in the Cortezian, Panama Bight and Cocos Island ecoregions, where species turnover is high across both latitudinal and longitudinal gradients. At the provincial level, we found that the contributions of the β-diversity from north to south, in the WTNP, TEaP and Gala were high, as result of the high number of unique species. Species turnover was also high at this scale, with a low contribution from species nestedness that was probably due to the low species/gene flow within the provinces. These results highlight the importance and successful inclusion of a cryptobenthic fish component in ecological and biogeographical studies.

## Introduction

One of the main topics in ecology is to understand the biogeographical patterns of species [1] and identify the drivers that determine the variation in biodiversity at different spatial scales [2,3]. This information can be used to understand the ecological importance of species that differentiate, characterize and preserve natural communities [4,5]. In reef fish, patterns of species diversity may differ, and they can be influenced by different drivers at different spatial and temporal scales [6], which may have an effect on the structuring of different fish assemblages from local to landscape or regional scales. The partitioning of species diversity *sensu* Crist et al. [4] is one of the most effective methods used to assess the variation in fish diversity [6], which allows for the relative contributions of local diversity (α-diversity), species turnover (β-diversity) and the combination of α- and β-diversity (γ-diversity) to landscape diversity to be quantified. The ecological meaning of β-diversity depends on the biodiversity partitioning approach. In additive partitioning, β-diversity quantifies the increase in diversity between the local and regional scale [7], while in multiplicative partitioning, β-diversity represents the effective number of distinct communities *sensu* Veech et al. [8], who estimated the difference in species composition at different spatial scales [7]. Therefore, the partitioning of γ-diversity allows for the quantification of the relative contributions of the α-diversity (local scale) and β-diversity (several scales) to the γ-diversity (additive approach), as well as the effective differentiation of the community at each spatial scale (multiplicative approach) [4]. Regardless of the processes that affect β-diversity, the result may be due to either the replacement or the loss/gain of species [9]. In this sense, additive β-diversity partitioning is used to estimate the relative contributions of replacement and nestedness species components [9,10].

Gobiidae *sensu* Gill and Mooi [11] is one of the most diverse fish families (~1,900 species), and is considered one of the most successful evolutionary lineages of vertebrates [12]. The success of the lineage is the result of extensive adaptive radiation that has allowed them to colonize mainly marine and coastal ecosystems, with a significant presence on oceanic islands [13]. Gobiidae contains five subfamilies [14,15] and more than 170 genera [16,17]. Subfamily Gobiinae tribe Gobiosomatini [18] is an endemic and diverse clade to the Americas, commonly called the "American seven-spined gobies", with 14 genera in the ETP and 52 species [19,20]. Despite previous research [21–34], our knowledge of the richness, diversity, distribution and biogeography of gobiid fishes in the ETP is still limited.

In this study, we assessed the γ-diversity partitioning of goby fish along a latitudinal (north-south) and longitudinal (islands-continent) gradient in the ETP. We hypothesized that γ-diversity can be divided into α and β components, with significant differences at the ecoregion and province levels that are greater than those at the sampling unit and realm levels. Moreover, we hypothesized that β-diversity could be explained more so by species turnover than nestendness species, and these explanations could mainly result from pairwise comparisons between insular-coastal ecoregions, since gobies are cryptobenthic, have close associations with the benthos and low geographic distribution in several species. Therefore, the objectives of the study were to (1) generate a complete checklist of marine and coastal goby fish species of the ETP. From the generated database and using the delimitation of the ecoregions, provinces and realms *sensu* Spalding et al. [35], the patterns of α- and β-diversity at different spatial scales were analysed; (2) Estimate (i) the relative contributions of α- and β-diversity to γ-diversity and (ii) the number of different communities or differentiation of the community at different spatial scales; and finally, (3) turnover (B_JTU_) and species loss/gain (B_JNE_) were determined at different spatial scales based on the results of community differentiation.

## Material and methods

### Study area and data collection

**Fig 1:**
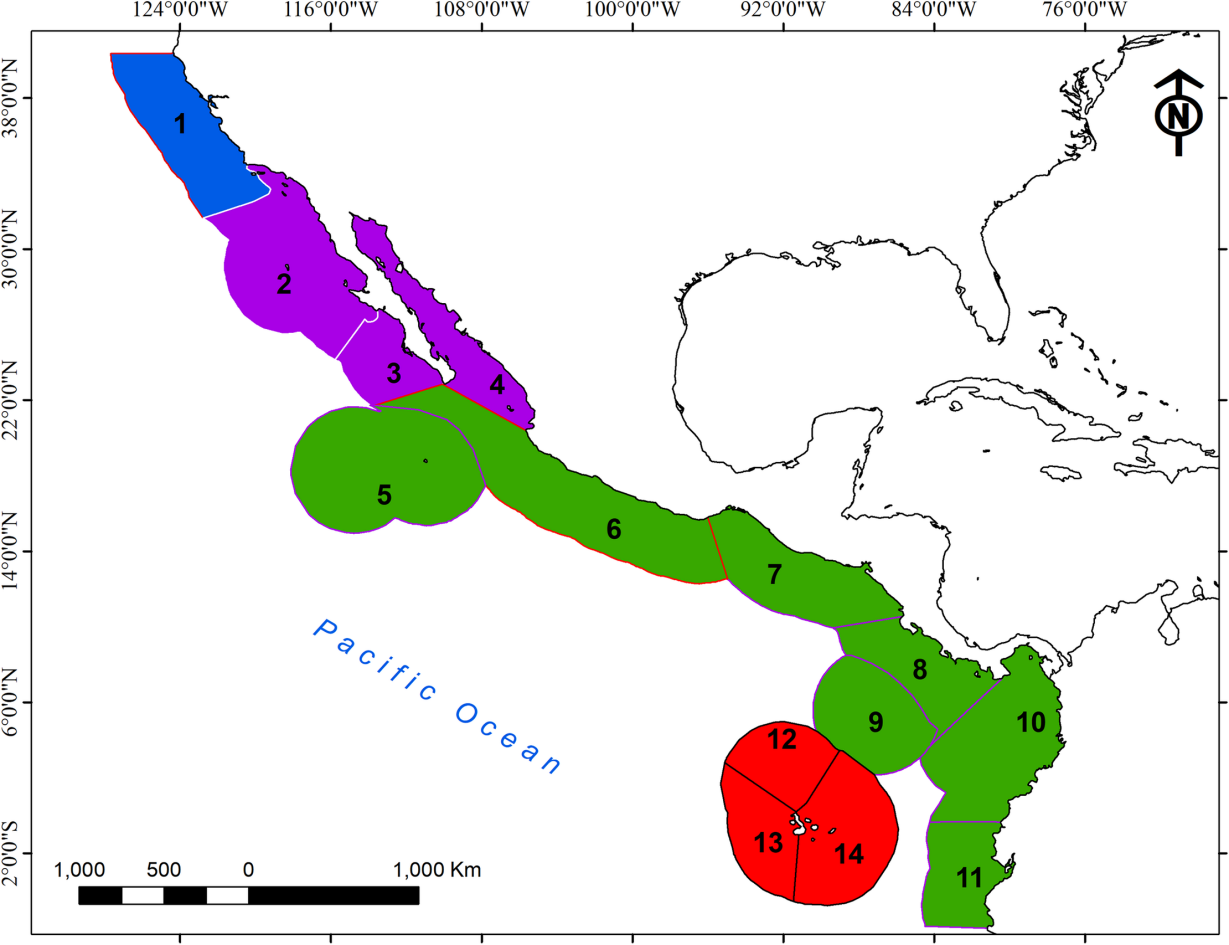
Map of study area divided in 14 ecoregions, four provinces and two realms. **Abbreviation are in brackets. Ecoregions**: 1 = Northern California (NCa), 2 = Southern California Bight (SCB), 3 = Magdalena Transition (MaT), 4 = Cortezian (Cor), 5 = Revillagigedos (Rev), 6 = Mexican Tropical Pacific (MTP), 7 = Chiapas-Nicaragua (CNi), 8 = Nicoya (Nic), 9 = Coco Island (CIs), 10 = Panama Bight (PaB),11 = Guayaquil (Gua), 12 = Northern Galapagos Islands (NGI), 13 = Western Galapagos Islands (WGI), 14 = Eastern Galapagos Islands (EGI). **Provinces**: Cold Temperate Northeast Pacific (CTNP; blue), Warm Temperate Northeast Pacific (WTNP; purple), Tropical East Pacific (TEaP; green), Galapagos (Gala; red). **Realms**: Temperate Northern Pacific (TNP; 1–4) and Eastern Tropical Pacific (ETP; 5–14). DOI: dx.doi.org/10.17504/protocols.io.sa5eag6.

The study area comprises the entire ETP *sensu* Roberson and Cramer [36] and Robertson and Allen [37], which extends from Magdalena Bay, Mexico (~ 24N, -112W) to northern Peru (~ 4S, -81W). However, to eliminate species border effects from the analysis, the complete distribution of the species present in the north was included (up to Tomales Point, California; ~ 38.2N, -122.9W), while the southern ETP is well delimited down to Guayaquil, Peru (Fig 1). Therefore, the area includes the entire ETP realm, and partially includes the Temperate Northern Pacific realm (TNP), following the marine regionalization by Spalding et al. [35]. The limits of the ETP [36,37] are demarcated by the turns of cold ocean currents, the proportion of endemism and the turnover of fauna to the northern and southern limits [38,39]. The ETP includes a complex diversity of coastal environments and oceanic islands (i.e. Gulf of California, Cocos Island, Gorgona and Galapagos) with important environmental variation [40], from very dry in the Gulf of California, southern Ecuador and most oceanic islands to very wet from Costa Rica to northern Ecuador, including Cocos Island [41]. The oceanographic conditions within the ETP vary seasonally, annually and over longer time scales, and these conditions are mainly influenced by three water masses: (i) subtropical surface water (sea surface temperature [SST]<25°C, salinity>35) that is located in the central turns of the North Pacific and south of the ETP; (ii) tropical surface water (SST>25°C, salinity<34) north of the equator, and (iii) equatorial surface water (SST<25°C, salinity>34) along the equator [42].The TNP is dominated by the oceanographic conditions of the California Current (SST<15°C, salinity<34) [43].

A database was constructed from four sources: (i) specimens collected from 2015 to 2017 from the ETP that were identified and deposited at the Colección de Peces, Universidad Michoacana (CPUM, MICH-PEC-227-07-09); (ii) review of material deposited in biological collections (Museo de Zoología, Universidad de Costa Rica, Colección Nacional de Peces, Universidad Nacional Autónoma de Mexico, and CPUM); (iii) occurrence data from the Global Biodiversity Information Facility [44]; and (iv) primary literature. All data were mapped in ArcMap 10.4.1 [45]. Data records with incorrect latitude and longitude or without scientific names were removed from the analysis (S1 Appendix).

Manipulation of fishes was carried out in strict accordance with the recommendations to the current law of Mexico NOM-062-ZOO-1999 [46], approved by the Divisional Council of Biological and Health Sciences of Universidad Autonoma Metropolitana (Universidad Autonoma Metropolitana, session 8.1, May 18, 2010).The organisms used in this study were lawfully obtained with the scientific collection permits for Mexico: PPF/DGOPA-035/15 and PPF/DGOPA-116/17; El Salvador: MARN-AIMA-004-2013; Costa Rica: SINAC-CUS-PF-R-122/2015 and R056-2015-OT-CONAGEBIO; and Panama: 78-Panamá; EC: 013/2012 PNG.

### Taxonomic identification and checklist

Taxonomic identification followed Van Tassell [47] and Robertson and Allen [37]. Additionally, we considered taxonomic revisions of the genera *Akko* by Van Tassell and Baldwin [34], *Aruma* by Hoese [48], *Barbulifer* by Hoese and Larson [49], *Bathygobius* by Miller and Stefanni [50], *Gobionellus* by Ginsburg [51], *Gobiosoma* and *Garmannia* by Ginsburg [52], *Gobulus* by Hoese and Reader [33], *Lythrypnus* and *Chriolepis* by Bussing [31] and *Elacatinus* by Hoese & Reader [32], and, in few cases, original descriptions. The systematic arrangement followed Van der Laan et al. [14] and Nelson et al. [53], and the taxonomic statuses of each species and genera were corroborated in Eschmeyer et al. [15]. The complete checklist included valid names and synonyms.

### Data analysis

Based on the bioregionalization of coastal and shelf areas proposed by Spalding et al. [35] for the eastern Pacific, a multiscale analysis of species richness and composition was performed. We employed Spalding et al. [35] approach since it represents a comprehensive biogeographic system to classify the oceans, particularly adequate for coastal and shelf waters, but also because it is hierarchical and nested and allow for multiscale analyses. We evaluated species richness and composition with an unbalanced nested hierarchical design at four spatial levels. The first level contained 159 sampling units (SUs) that correspond to 1° latitude x 1° longitude cells. These SUs were grouped into 14 ecoregions (2^nd^ level), four biogeographic provinces (3^rd^ level) and two realms (4^th^ level) (S1 Fig). In Fig 1, we provide complete information about acronyms.

To evaluate the representativeness of the sampling effort and recorded biological inventory, sample-based rarefaction curves were constructed based on the observed richness (S_obs_) for each SU, while the expected richness was calculated with the non-parametric estimators Chao 2, Jackknife 1, Jackknife 2 and Incidence Coverage Estimator (ICE).

To evaluate the spatial affinities of the composition of the gobiid fish ensemble from the ETP, a principal coordinates ordination (PCO) analysis was performed using Jaccard’s similarity matrix. To estimate the relative importance of α- and β-diversity at different spatial scales and their contributions to the overall diversity (γ-diversity), we estimated (i) the additive diversity partitioning, which is used to calculate the contribution of α- and β- diversity to γ-diversity [8]; (ii) multiplicative diversity partitioning, which is used to identify the effective number of completely distinct communities (i.e. species turnover rates) at each spatial scale [54]; and (iii) several β-diversity partitions *sensu* Baselga [9] to evaluate whether the β-diversity patterns are the results of species turnover or nestedness (species loss/gain) at different spatial scales. The partitioning analyses were performed with an incidence matrix (i.e. presence/absence).

In the additive diversity partitioning, the relative contributions of α-diversity and β-diversity with respect to γ-diversity were estimated. This procedure considered the average species richness of gobiid fishes per sampling unit as the local diversity (ᾱ-diversity) and the species turnover (β-diversity) among SUs (β_add1_), ecoregions (β_add2_), provinces (β_add3_) and realms (β_add4_). This spatial variation design was created according to Veech et al. [8]. Therefore, the utilized design was:

$$\gamma -\mathrm{diversity}={\bar{\alpha }}_{\left(\mathrm{SU}\right)}+\beta_{\mathrm{add}1\left(\mathrm{SU}\right)}+\beta_{\mathrm{add}2\left(\mathrm{ecoregions}\right)}+\beta_{\mathrm{add}3\left(\mathrm{provinces}\right)}{+\beta }_{\mathrm{add}4\left(\mathrm{realms}\right)}$$ (1)

In the multiplicative diversity partitioning approach (β_mult_), α-diversity is the average diversity found in a single randomly chosen sample, and β-diversity is the effective number of completely distinct communities in terms of species composition at each level. Therefore, the value of β is unity (1) when all communities are identical in their species composition, and N (number of communities) when all communities are completely distinct from each other in terms of shared species [7]. In this way, β_mult1_ (1/159) ranged from 1–159 at the local scale, β_mult2_ (1/14) ranged from 1–14 at the ecoregion level, β_mult3_ (1/4) ranged from 1–4 at the province level, and β_mult4_ (1/2) ranged from 1–2 at the realm scale. Therefore, the utilized design was:

$$\gamma -\mathrm{diversity}={\bar{\alpha }}_{\left(\mathrm{SU}\right)}+\beta_{\mathrm{mult}1\left(\mathrm{SU}\right)}\;x\;\beta_{\mathrm{mult}2\left(\mathrm{ecoregions}\right)}\;x\;\beta_{\mathrm{mult}3\left(\mathrm{provinces}\right)}{\;x\;\beta }_{\mathrm{mult}4\left(\mathrm{realms}\right)}$$ (2)

The additive and multiplicative partitions were constructed based on the samples (incidence data) with unweighted SUs in an unbalanced design. Unrestricted, sample-based randomization was used. We used the Hill number of order q = 0, which expresses the effective number of different elements and is equally sensitive to rare and common species [54]. Null models with 10,000 randomizations per spatial level were constructed to evaluate the statistical significance of the observed *vs*. expected α- and β-diversity. However, due to the use of incidence data and sampled-based incidence, it was not possible to estimate the expected values and evaluate the statistical significance of the lowest spatial level [55], ᾱ_(SU)_, β_add1_ and β_mult1_.

Baselga's [9] β partitioning considers the overall β-diversity (e.g., Jaccard dissimilarity), which can be additively divided into two components that represent the spatial turnover in species composition (B_JTU_) and the variation in species composition because of nestedness (B_JNE_). We calculated β-diversity partitioning for scales in which the β_mult_ values were significantly different. To complement this analysis and demonstrate the species that contributed most to species turnover, shared and unshared species were identified at these spatial scales. We consider both unique (appear in only one sample) and duplicate (two samples) species as unshared species, while shared species were found in three or more samples.

Finally, sample-based curves were constructed with 10,000 non-replacement randomizations in ESTIMATES 9.1 [56]. The PCO was carried out in PRIMER 6.1 PERMANOVA+ v.1.0.6 [57,58]. The additive and multiplicative diversity partitioning analyses were carried out using PARTITION 3.0 [55], while β-diversity partitioning (using Jaccard dissimilarity family) was performed with the “betapart” package [59] in R-project software [60].

## Results

### Sample effort, species richness and spatial analysis

**Fig 2:**
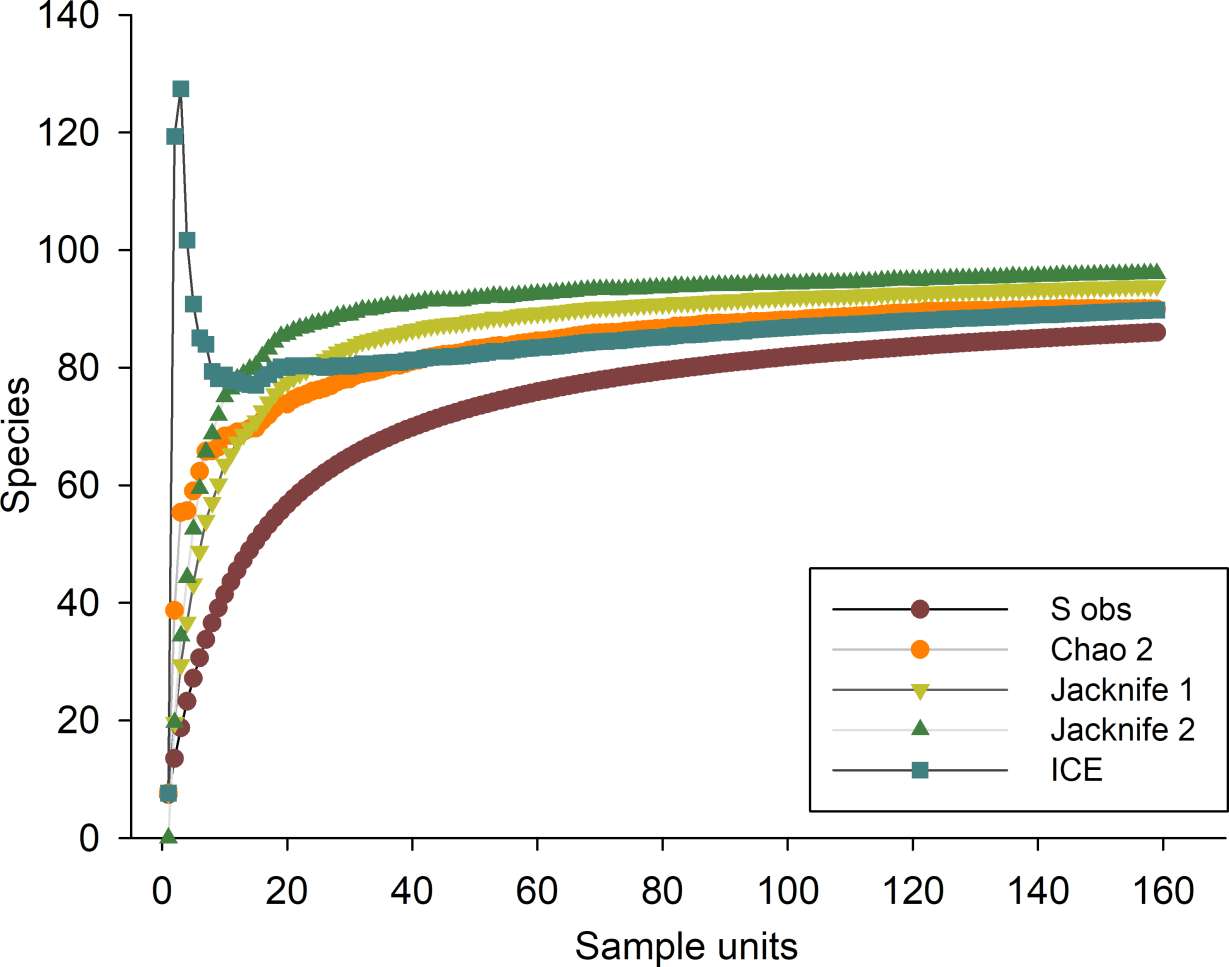
Sampled-based rarefaction curves of observed (S_obs_) and expected gobies species richness. Expected richness estimated by non-parametric procedures (Chao 2, Jackknife 1, Jackknife 2 and Incidence Coverage Estimator (ICE). Curves were constructed with 10,000 non-replacement randomizations. DOI: dx.doi.org/10.17504/protocols.io.sbdeai6.

The sample-based rarefactions curves showed that the observed richness (87 species) tended to reach an asymptote, with a representativeness of 93% with respect to the average value of the non-parametric estimators (Chao 2, Jackknife 1, Jackknife 2 and ICE). The highest estimate of expected richness was obtained by Jackknife 2 (96 species), while the lowest estimates were obtained by ICE and Chao 2 (90 species each). These results confirmed an adequate sampling effort of the biological inventory of the gobies from the ETP (Fig 2).

The database included a total of 8,525 records of gobies from field collections, museums and ichthyological collections, open access databases, and the literature. The updated checklist of species from the ETP consisted of 87 valid species distributed in 27 genera and two subfamilies (S1 Checklist); *Awaous transandeanus* was included in the checklist, but it was excluded from the subsequent overall analyses due to their serious taxonomic inconsistencies (see Discussion). The Gobiidae tribe Gobiosomatini was composed of 22 genera and 67 species, which represents 77% of the species from the ETP. The richest genera were *Lythrypnus* (10 species), *Gobiosoma* and *Microgobius* (9 species each). The best-represented species were the intertidal goby *Bathygobius ramosus* (12.5% of the total records), followed by the sand-rubble goby *Coryphopterus urospilus* (9.2%), the muddy goby *Quietula y-cauda* (8.6%) and the rocky-reef goby *Elacatinus puncticulatus* (8.2%), which together made up 38.5% of the total records (see S1 Appendix). The best-represented ecoregions were Panamá (54 species), Cortezian (50), Nicoya (38) and Chiapas-Nicaragua (37). At province level, were TEaP (71 species) and WTNP (51); while at realms level were ETP (73 species) followed by TNP (51) (S1 Table).

**Fig 3:**
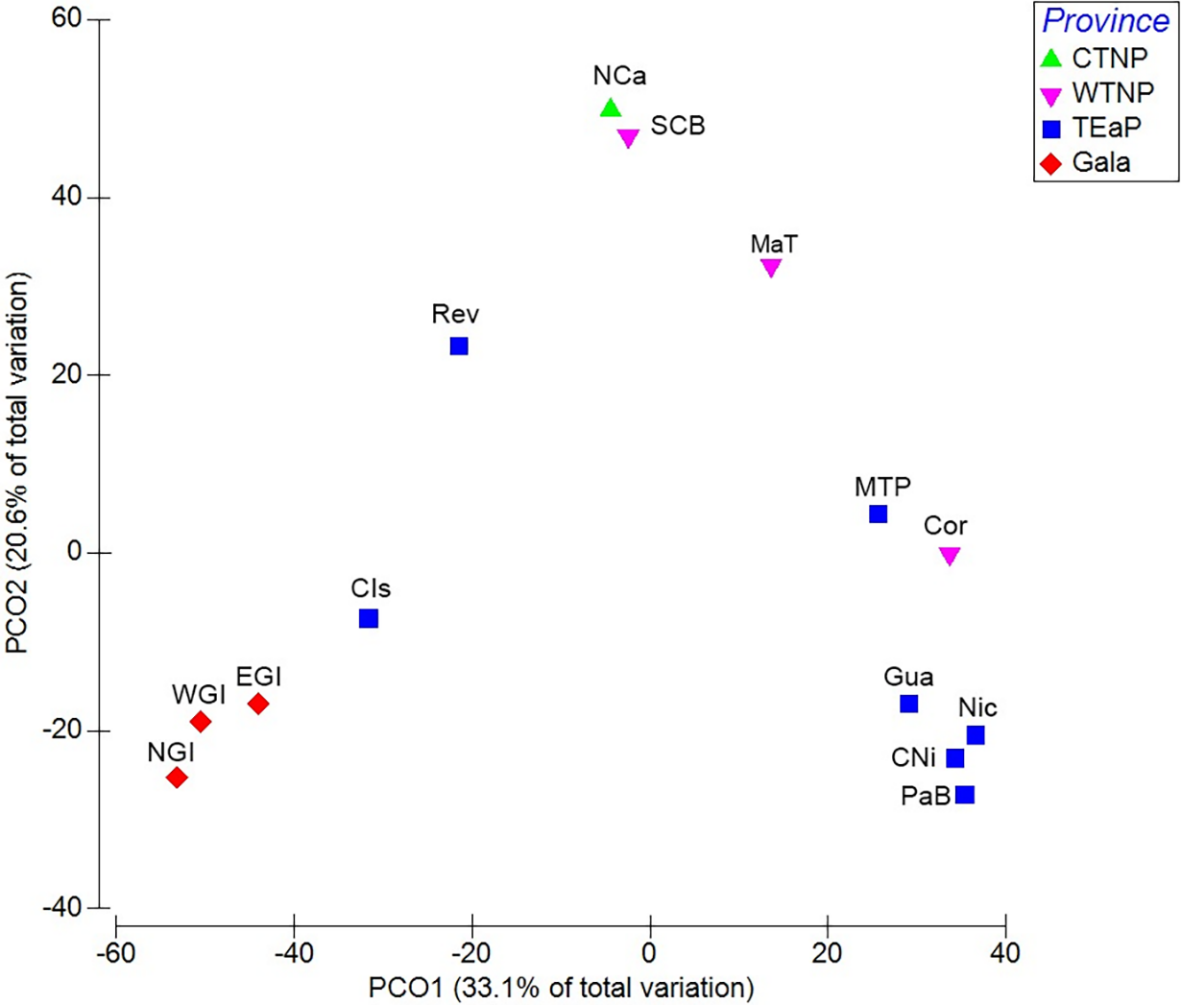
PCO plot based on gobies fish incidence data. **All marine ecoregions and provinces were grouped *sensu* Spalding et al. [35]. Jaccard similarity was used as a resemblance measure of species composition.** Ecoregions and provinces abbreviations are shown in Fig 1. DOI: dx.doi.org/10.17504/protocols.io.sbceaiw.

The two orthogonal components in the PCO analysis explained 53.7% of the total variation. The first component (PCO1 = 33.1%) explained the variation in species composition between coastal ecoregions and island ecoregions (longitudinal gradient), while the second component (PCO2 = 20.6%) was related to the species replacement in the species composition along the latitudinal gradient (Fig 3). In both orthogonal components, the variation in the goby ensemble resulted from the strong differences across the latitudinal and longitudinal gradients between ecoregions.

### γ-diversity

In the additive partitioning of goby diversity, the β-diversities between ecoregions (β_add2_) and provinces (β_add3_) were higher than expected from the null models, while the β_add4_ values between realms were lower than expected from the null models. At the landscape level, ᾱ_SU_ contributed with 7 species (8.1% of total richness), β_add1_ contributed with 14 species (16.3%), β_add2_ contributed with 14 species (16.3%), β_add3_ contributed with 27 species (31.49%) and β_add4_ contributed with 24 species (27.9%) (Table 1). Otherwise, the multiplicative partitioning evidenced that the β-diversity between ecoregions (β_mult2_) and provinces (β_mult3_) was statistically significant with respect to the null models; therefore, at these spatial levels, there was an effective species turnover of the goby fish community (Table 1). At the lowest level (β_mult1_), there were only three different goby fish communities. Considering that the probable number is 159 communities (expected), there were two distinct communities (2/14) at ecoregion level (β_mult2_), two communities (2/4) at the province level (β_mult3_), and only one community (1/2) at the landscape level (β_mult4_).

**Table 1:** Results of the additive and multiplicative diversity partitioning of gobiid fishes.

	Spatial level	Observed values	Expected values	
			Mean	Intervals
**Additive partition**				
	ᾱ	7.4	*nd*	*nd*
	*β*_add1_	13.6	*nd*	*nd*
	*β*_add2_	13.5*	5.68	2.57–9.5
	*β*_add3_	27.5*	18.32	13.75–27.5
	*β*_add4_	24	31.46	24–46.5
**Multiplicative partition**				
	ᾱ	7.4	*nd*	*nd*
	*Β*_mult1_	2.84	*nd*	*nd*
	*Β*_mult2_	1.64*	1.2	1.08–1.38
	*Β*_mult3_	1.8*	1.43	1.28–1.8
	*Β*_mult4_	1.39	1.56	1.39–1.9

Expected interval is the minimum and maximum values produced by an expected distribution of the diversity components from which *p*-values are obtained under null models built by 10,000 randomizations.

* **=** statistical significance (*p*≤0.001). *nd* = no data. Sample-based randomization cannot be applied to the lowest level of analysis (level 1); thus, no statistical null distribution was produced to this level.

DOI: dx.doi.org/10.17504/protocols.io.s3iegke

### β-diversity

**Fig 4:**
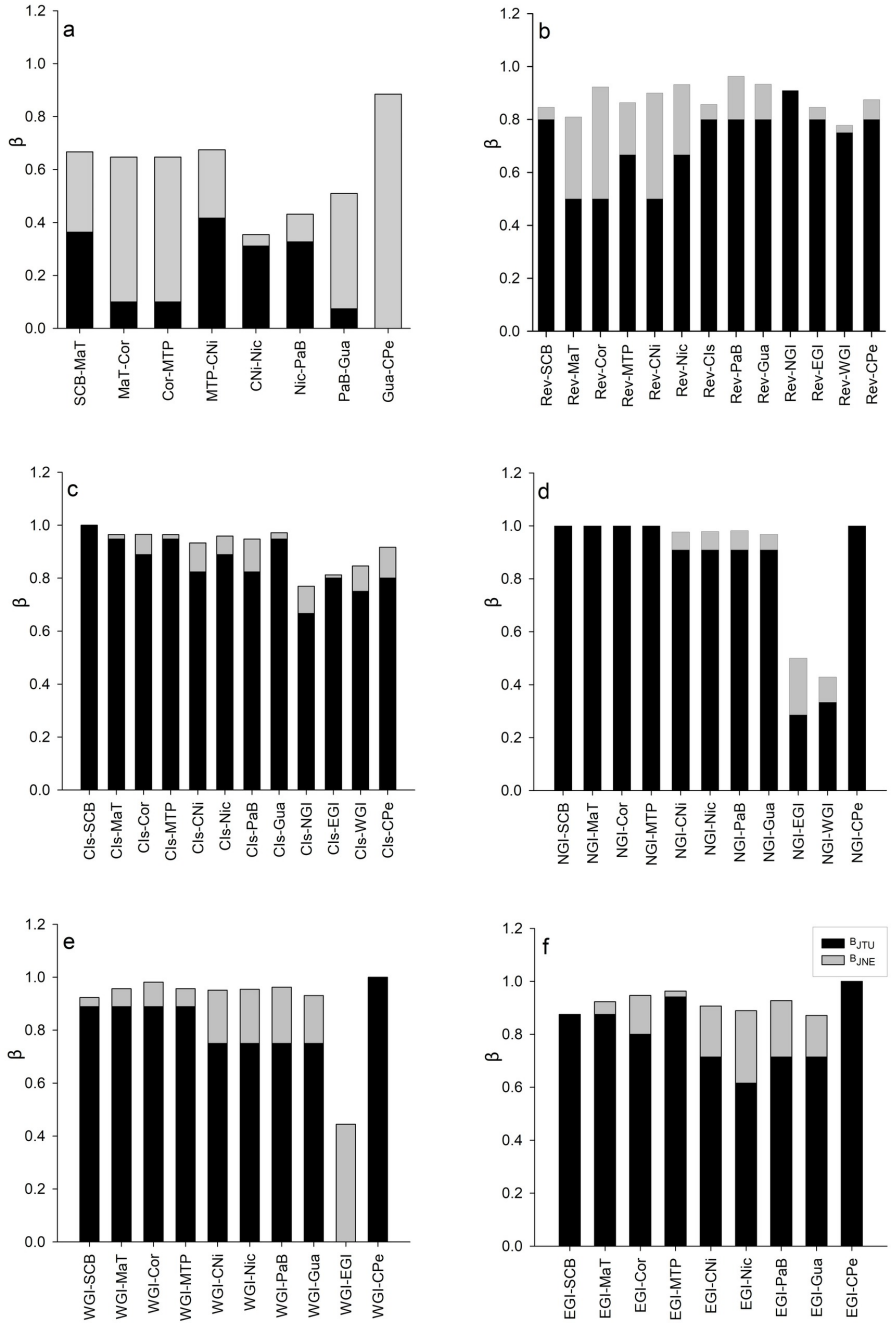
Evaluation of β-diversity partitioning in its spatial species turnover (B_JTU_) and nestedness (B_JNE_) components along a latitudinal gradient across biogeographic ecoregions. (a) Coastal ecoregions, and islands ecoregions versus all ecoregions, (b) Revillagigedo, (c) Coco Island, (d) Northern Galapagos Island, (e) Western Galapagos Island and (f) Eastern Galapagos Island. DOI: dx.doi.org/10.17504/protocols.io.sbfeajn.

The results from the γ-diversity partitioning (βmult) showed statistically significant differences at both the ecoregion and province spatial scales. In this regard, we computed the β-diversity at these levels to determine if the community differentiation was due to species replacement (B_JTU_) or species loss/gain (B_JNE_). In general, the species turnover was higher (B_JTU_ = 0.813) than nesting (B_JNE_ = 0.115) between ecoregions (S2 Table). Considering only the coastal ecoregions in a latitudinal gradient (from north to south), nesting was significantly higher (B_JNE_ = 0.297) than species replacement (B_JTU_ = 0.274; Fig 4A). However, pairwise comparisons showed that species replacement played predominant roles in the north (between the NCa-SCB) [B_JTU_ = 0.5; B_JNE_ = 0.136] and the SCB-MaT [B_JTU_ = 0.364; B_JNE_ = 0.303]) and the south (between CNi-Nic) [B_JTU_ = 0.31; B_JNE_ = 0.04], Nic-PaB [B_JTU_ = 0.32; B_JNE_ = 0.10]) of the study area (S2 Table). In addition, the richness of species did not come from a similar proportion along latitudinal gradient (χ^2^ = 78.29, df = 8, p<0.0001).

The analysis of the insular ecoregions vs. the rest of the ecoregions showed that the species diversity was very high in the insular ecoregions (S2 Table). Cocos Island maintained a high species replacement on average (B_JTU_ = 0.866, B_JNE_ = 0.056), although low values of species nesting in adjacent ecoregions (i.e. CNi, Nic, PaB) were obtained (Fig 4C). The Galapagos archipelago ecoregions maintained high average replacement values (NGI, [B_JTU_ = 0.841, B_JNE_ = 0.053], WGI [B_JTU_ = 0.84, B_JNE_ = 0.118], EGI, [B_JTU_ = 0.802, B_JNE_ = 0.114]), mainly with the northern ecoregions (NCa, SCB, MaT, Cor, MTP and Rev). Otherwise, low nesting values were obtained in the southern ecoregions (CNi, Nic, PaB, Gua and CIs), but they increased in the contiguous ecoregions (Fig 4D and 4F). High species turnover was maintained to the north and south of Revillagigedo (Fig 4B), and on average, was greater than the nesting (B_JTU_ = 0.715, B_JNE_ = 0.159); however, the turnover in Revillagigedo was relatively lower than that in nearby continental ecoregions (i.e. MaT, Cor, MTP, CNi). In all cases, the relatively low nestedness values could suggest the existence of relatively more permeable borders to the movement of species/gene flow between ecoregions. In this case, the richness of species remained at similar proportions among insular ecoregions (χ^2^ = 2.61, df = 4, p<0.624). The number of unshared species in the ecoregions was high and constituted 41.9% (uniques = 26 species, duplicates = 10 species) of the total species, and these species occurred in the Corteziana (14 species), Panama Bight (13), Cocos Island (5) and Nicoya (4) ecoregions (S3 Table).

**Fig 5:**
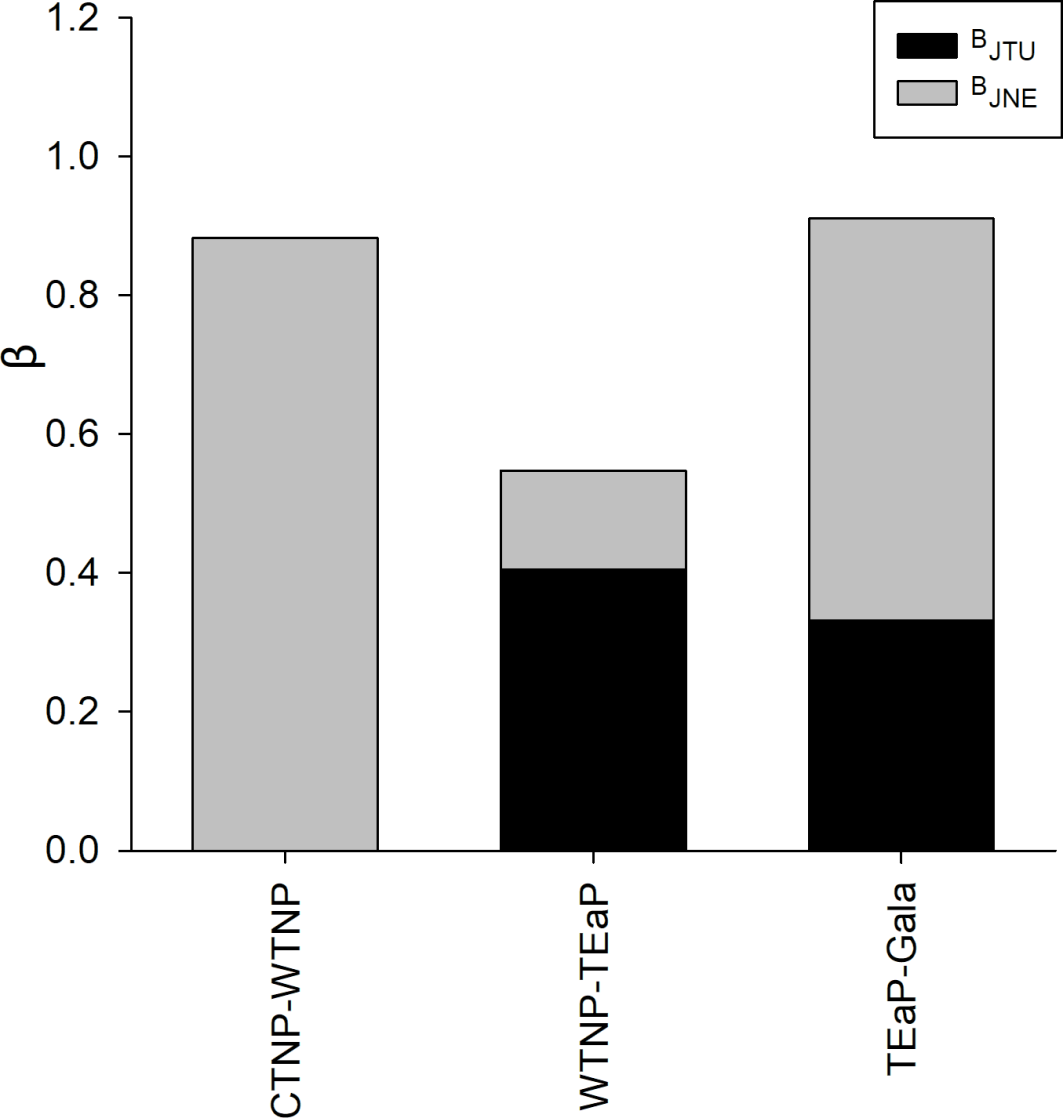
Evaluation of β-diversity partitioning in its spatial turnover (B_JTU_) and nestedness (B_JNE_) components along a latitudinal gradient across biogeographic provinces. Abbreviations are showed in Fig 1. DOI: dx.doi.org/10.17504/protocols.io.sbbeain.

At the province spatial scale, the overall analysis showed that the species turnover (B_JTU_ = 0.527) was greater than the nestedness (B_JNE_ = 0.322) (S4 Table). Over a latitudinal gradient between provinces, the nestedness of the CTNP vs. WTNP was 100% (B_JNE_ = 0.882); however, between the WTNP and TEaP species turnover (B_JTU_ = 0.406) was greater than the nestedness (B_JNE_ = 0.141). Between the TEaP and Gala, nestedness was higher (B_JTU_ = 0.333, B_JNE_ = 0.557) (Fig 5). At the provincial level, the number of unshared species was higher, and shared species constituted 93.0% of the total species (uniqueness = 41 species, duplicates = 39 species), while six species were shared species (S3 Table). The species richness among provinces did not come from similar richness proportions (χ^2^ = 87.45, df = 3, p<0.0001).

## Discussion

An updated checklist with 87 valid species of marine and coastal goby fishes of the ETP is presented in this study. *Awaous transandeanus* is cautiously included in the checklist but was excluded from the subsequent diversity analyses due to the serious taxonomic inconsistencies between *A*. *transandeanus* and *A*. *banana*, and the fact that their geographical distributions are not yet adequately delimited (see [61]). Otherwise, the checklist includes the genera *Elacatinus* and *Tigrigobius sensu stricto* Rüber et al. [13] and Tornabene & Van Tassell [20]. In the genus *Tigrigobius*, the species *Tigrigobius digueti* and *T*. *inornatus* were considered valid based on the works of Taylor and Hellberg [62] and Robertson and Allen [37] (S2 Fig). In addition, we cautiously considered the records of *Bathygobius lineatus* for Costa Rica, Colombia and Peru because Robertson and Allen [37] considered it an endemic species of the Galapagos.

The sampling effort was represented by 87 species, which explains 93% of the total expected species richness according to the non-parametric estimators, and indicates that the inventory of gobies from the ETP was correctly sampled. The relatively high species richness that was recorded resulted from the conjunction of different information sources (i.e. field sampling, use of biological collections/museums, open access databases and literature). However, there are still large areas and different environments in the ETP that must be systematically surveyed to obtain complete biological inventories. Our work and other similar studies [63–65] have found gaps in the biological information for gobies in northwestern Mexico (Sinaloan Gap, Marias archipelago, Marietas and Isabel Island), the Revillagigedo archipelago, Central America and Colombia that need to be more adequately sampled.

### γ-diversity partitioning

The goby diversity partitioning shows that β-diversity (additive and multiplicative approach) is the most important component that explained the γ-diversity. The additive and multiplicative partitions suggest that at the sampling unit and realm levels, the diversity is homogeneous and there is no differentiation in the community. The greatest variation was obtained at the ecoregion and province levels, suggesting that at these levels, β-diversity explained the most variation in the diversity of gobiid fishes in the ETP, as well as the structure of the gobiid fish ensembles at both scales. At the ecoregion level, β-diversity could be influenced by the life histories of gobies. For example, several species are habitat specialists with specific microhabitat requirements [66–68] or live in obligate association with hosts such as sponges [62,69], hydrozoans, corals and octocorals [66,70] and alpheid shrimps [16,71]. Goatley et al. [72] and Coker et al. [73] suggested that cryptic fish ensembles (including gobiid fishes) exhibit fine-scale partitioning that is determined by the local patterns and processes such as substrate dependence, high endemism and speciation, which are products of a complex evolutionary history that is emphasized at the regional level [74,75]. In the Caribbean, Rodríguez-Zaragoza et al. [6] in the central Mexican Pacific and Acosta-González et al. [76] and Francisco-Ramos & Arias-González [77] found that the most important variation in the structure of benthic fish communities at the local scale is due to β-diversity, which is explained by several factors such as the species-substrate association, habitat complexity (geomorphology), environmental variables, and the rarity of species. These findings could be related to our results because the inventory was confirmed by a high number of rare species (41.9%, uniques = 26 species, duplicates = 10 species) mainly from the Cortezian, Panama Bight and Cocos Island ecoregions. Uniques/duplicates are indicative of the restricted geographical distribution of species, and these species are generally endemic or difficult to register in biological inventories [78,79] and play a predominant role in the structuring of local and regional assemblages [80,81]. Violle et al. [82] considered rare species as "atypical ecological" species because of their taxonomic rarity at a local scale and because of their functional exclusivity and taxonomic restriction on a regional scale. Within the ETP, the rarity of gobies between ecoregions was related to high endemism. For example, *Gillichthys detrusus* is endemic to the Colorado River delta, the *Lophogobius* genus is restricted to the Panama Bight, while *Lythrypnus alphigena*, *L*. *lavenbergi* and *L*. *cobalus* are endemic to Cocos Island.

At the province level, additive and multiplicative components (β_add3_ and β_mult3_) had the greatest contribution to γ-diversity. Therefore, the province level was the most important spatial level to explain the patterns in the variation in composition, diversity, distribution and life history (biogeography) of the ETP gobies. The additive partition contributed 32% to the γ-diversity, while the multiplicative partition showed high community differentiation (1.8/4). Francisco-Ramos & Arias-González [77] suggest that at broad geographic scales (i.e. provinces), the contribution of β-diversity was influenced by not only the current connectivity of species, but also by important biogeographical, evolutionary and ecological components (i.e. isolation, barriers, island size and habitat diversity) that operate at varying spatial scales. Moreover, the contribution of β-diversity is also influenced by oceanographic and tectonic processes on a regional scale that limit gene flow and promote the differentiation of species/ensembles between regions [36,83]. Rodríguez-Zaragoza et al. [6] suggest that the structural elements of habitat also influence mesoscale processes and, consequently, this increases the availability of ecological niches, and the richness and rarity of species simultaneously increase. Here, we found a high number of unshared species at the province level (93%, uniqueness = 41 species, duplicates = 39 species) in the ETP; this indicates a high number of rare species, and suggests a low connectivity in the genes/species flow between provinces.

### β-partitioning

The partition of the β-diversity from coastal marine ecoregions over a latitudinal gradient showed that there is high species turnover in the northern (NCa, SCB) and southern (CNi, Nic, PaB and Gua) parts of the study area. Moreover, the nesting of species is high in the central ecoregions (MaT, Cor and MTP), indicating that the ensemble structure of coastal gobies differs along the latitudinal gradient. Various biogeographical classifications of the Eastern Pacific (i.e. [36,63,84,85]) have shown that fish composition varies along latitudinal gradients, which is a result of an intricate geological and biogeographic history of the ETP [86,87]. This history has promoted areas of high endemism such as the Gulf of California, Panama Bight and oceanic islands. The results of this study corroborate that in the coastal zone of the ETP, there are two areas of high endemism of gobiid fishes: Cortezian with 14 species and the Panama Bight with 13 species (S1 Appendix). Our results partially agree with the results of Hastings [63], who identified two areas of high endemism in chaenopsid fishes, Cortezian and Panamic provinces, which are separated by the Central America Gap and the Sinaloan Gap *sensu* Briggs [84], which limits the dispersal of species due to wide extents of sandy bottoms.

The overall results of the β-diversity of the insular *vs*. continental ecoregions indicate that there a high replacement of species. This result confirms that insular and coastal goby ensembles are distinct and high endemism is preserved (i.e. Cocos Island). Hastings [63] suggested that the high differentiation in the richness and composition of insular and continental chaenopsid fishes in the ETP are due to the closure of the Isthmus of Panama and the genetic fragmentation of insular and coastal species by allopatry [13]. This process allowed for the isolation of the reef fish fauna of the Caribbean Sea and the ETP over the last 3.2 million years [88–90]; at the same time, this allowed for the isolation from the Central Pacific by a physical barrier of approximately 5,000 km [91], which has resulted in high speciation, especially in some genera of gobies [62,92]. Thus, the current fauna of the ETP remains isolated from the Indo-Pacific and Central Pacific and is still strongly influenced by some species from the Caribbean which have crossed the Panama Canal (e.g. *Gobisoma hildebrandi*, *G*. *nudum*, *Barbulifer ceuthoecus*, *Lophogobius cyprinoides*) and that show a strong common evolutionary history [32,62]. Also, we found a relatively low values of species nesting that were observed among some insular ecoregions and neighbouring ecoregions (i.e. Rev—MaT, Cor and MTP; CIs—Nic and PaB) suggest that the borders to the exchange of genes/species between ecoregions are permeable, which is probably due to the prevailing oceanographic currents [93,94]. An example is the Rev ecoregion with respect to the MaT and Cor ecoregions, which was previously considered a critical route to connect the Revillagigedo Islands with the entrance of the Gulf of California and the Central Mexican Pacific for coral reef propagules [83].

At the provincial level, there is a marked differentiation in the species replacement and species nesting over a latitudinal gradient using pairwise comparison. Our results suggest that the goby ensemble of CTNP is completely nested in the WTNP province. Subsequently, the WTNP has a high species turnover with respect to the TEaP, while the TEaP and Gala have high species nestedness with a significant percentage of species turnover. This result indicates that the goby fauna from the WTNP, TEaP and Gala provinces are unique ensembles from each province, although they maintain an important species flow between provinces and especially wide distributions of species such as *B*. *ramosus*, *C*. *urospilus* and *T*. *puncticulatus*. This result coincides with the results of Hastings [63] and Robertson & Cramer [36], who identified three provinces for the ETP based on endemism (Cortezian, Panamic and Oceanic Islands), and they assumed important flows of species among these provinces. In our study that analysed the turnover of goby fish species across the ETP, the provinces in Robertson & Cramer [36] correspond to the WTNP ≈ Cortez, TEaP ≈ Panamic and Gala + CIs ≈ Oceanic Islands.

In summary, the overall results of this study show that the γ-diversity of gobiid fishes from the ETP is the result of the contribution of the β-diversity components at the ecoregion and province scales. Moreover, depending on the spatial scale, the latitudinal position and type of environment (i.e. coastal or oceanic), the contributions of species turnover and nestedness vary with the variationin total β-diversity. At both spatial scales, β-diversity is probably determined by the rarity of species and the life histories of interacting species (biogeographic history and speciation) and to a lesser degree by the oceanographic conditions and the physical barriers of the ETP. Lessios and Baums [93] showed that the continuous flow of genes and potentially the continuous flow of species between coastal and insular areas of the ETP are possible (i.e. reef fishes, corals and echinoderms). The success of larval settlement and connectivity depend on the pelagic larval duration (PLD) in the water column and the ontogeny of the species. In gobies, the PLD is short, and their propagules do not reach to colonize distant areas, other species live in strict association with other species, and species that undergo high speciation may be exposed to rapid extinction events [95,96]. Therefore, several marine gobies may be among the most threatened reef fish species [67]. Moreover, the great coastal and insular-oceanic environmental and oceanographic variability [87], as well as palaeontological, biogeographic [84], phylogenetic and evolutionary processes [62,63,97] have moulded the goby fish assemblages in the ETP, which allowed for the detection of a particular distribution pattern at each spatial scale that was studied, especially within and between the ecoregions of the Gulf of California, Panama Bight, Cocos Island and Galapagos Islands. Finally, we consider that the inclusion of the crypto-benthic fish ensemble as in this study, provides relevant ecological-biogeographical patterns due to its ecological requirements (i.e. cryptic nature, low dispersal and low colonizing capacities) and utilization of a particular ecological niche [98, 99]; and hence this ensemble could potentially be considered an excellent indicator group that reflects the global variation in marine biodiversity within and along the ETP.

## Supporting information

S1 Checklist Update checklist of gobies from Eastern Tropical Pacific with synonyms and records from literature. Habitat, m = marine, e = estuarine, fw = freshwater, br = brackish. Source, *revised species from collected specimens or from museums and fish collections, **obtained from GBIF or literature. Literature: 1 = Abbott (1989); 2 = Alzate et al. (2012); 3 = Alzate et al. (2014), 4 = Béarez, P. (1996), 5 = Béarez et al. (2007), 6 = Castellanos‐Galindo et al. (2005); 7 = Castellanos-Galindo & Krumme (2013); 8 = Castellanos-Galindo et al. (2014); 9 = Cortés (2012); 10 = De la Cruz-Agüero et al. (1994); 11 = Del Moral-Flores et al. (2013); 12 = Del Moral-Flores et al. (2016); 13 = Del Moral Flores et al. (2017); 14 = Díaz-Ruiz et al. (2004); 15 = Erisman et al. (2011); 16 = Fourriere et al. (2016); 17 = Fourriere et al. (2017); 18 = Galván-Villa et al. (2016); 19 = González-Murcia et al. (2012); 20 = Graham (1975); 21 = Hooker (2009); 22 = López & Bussing (1982); 23 = Martínez-Muñoz et al. (2016); 24 = Murase et al. (2014); 25 = Palacios-Salgado et al. (2012); 26 = Palacios-Salgado et al. (2014); 27 = Salas et al. (2015); 28 = Shervette et al. (2007); 29 = Tavera & Rojas-Vélez (2017); 30 = Torres-Hernández et al. (2016); 31 = Tornabene et al. (2012); 32 = Van der Heiden & Findley (1988); 33 = Villareal-Cavazos et al. (2000).
(DOCX)

S1 Appendix Database of gobies from Eastern Tropical Pacific with information of collected specimens, museums/fish collections, open access database, and literature. (XLSX)

S1 Fig Following to Spalding et al. (2007), gobies distribution was grouped in 159 sampling units (First level, L1), 14 ecoregions (Second level, L2), 4 provinces (Third level, L3) and 2 realms (Fourth level, L4). **Ecoregions abbreviations are in Fig 1. Provinces**: Cold Temperate Northeast Pacific (CTNP), Warm Temperate Northeast Pacific (WTNP), Tropical East Pacific (TEaP), Galapagos (Gala). **Realms**: Temperate Northern Pacific (TNP), Eastern Tropical Pacific (ETP).
(DOCX)

S2 Fig Distribution of *Tigrigobius digueti* (red), *Tigrigobius inornatus* (blue) and *Tigrigobius nesiotes* (green) in Eastern Tropical Pacific. (DOCX)

S1 Table Number of genera and species in each ecoregion, province and realms. **Ecoregions**: 1 = Northern California (NCa), 2 = Southern California Bight (SCB), 3 = Magdalena Transition (MaT), 4 = Cortezian (Cor), 5 = Revillagigedos (Rev), 6 = Mexican Tropical Pacific (MTP), 7 = Chiapas-Nicaragua (CNi), 8 = Nicoya (Nic), 9 = Coco Island (CIs), 10 = Panama Bight (PaB),11 = Guayaquil (Gua), 12 = Northern Galapagos Islands (NGI), 13 = Western Galapagos Islands (WGI), 14 = Eastern Galapagos Islands (EGI). **Provinces**: Cold Temperate Northeast Pacific (CTNP), Warm Temperate Northeast Pacific (WTNP), Tropical East Pacific (TEaP), Galapagos (Gala). **Realms**: Temperate Northern Pacific (TNP) and Eastern Tropical Pacific (ETP).
(DOCX)

S2 Table Beta diversity partitioning outputs of species turnover and nestedness components among ecoregions. (DOCX)

S3 Table Shared (S) and unshared (U/D = uniques/duplicate) species of goby fish per ecoregion and province level at Eastern Tropical Pacific. (DOCX)

S4 Table Beta diversity partitioning outputs of species turnover and nestedness components among biogeographic provinces. (DOCX)
