# Supplementary material for: Gamma-diversity partitioning of gobiid fishes (Teleostei: Gobiidae) ensemble along of Eastern Tropical Pacific: Biological inventory, latitudinal variation and species turnover
Source: PLoS One. 2018 Aug 31;13(8):e0202863. doi: 10.1371/journal.pone.0202863 (PMC6118385; doi:10.1371/journal.pone.0202863)
Supplement: S1 Checklist — Habitat, m = marine, e = estuarine, fw = freshwater, br = brackish. Source, *revised species from collected specimens or from museums and fish collections, **obtained from GBIF or literature. Literature: 1 = Abbott (1989); 2 = Alzate et al. (2012); 3 = Alzate et al. (2014), 4 = Béarez, P. (1996), 5 = Béarez et al. (2007), 6 = Castellanos‐Galindo et al. (2005); 7 = Castellanos-Galindo & Krumme (2013); 8 = Castellanos-Galindo et al. (2014); 9 = Cortés (2012); 10 = De la Cruz-Agüero et al. (1994); 11 = Del Moral-Flores et al. (2013); 12 = Del Moral-Flores et al. (2016); 13 = Del Moral Flores et al. (2017); 14 = Díaz-Ruiz et al. (2004); 15 = Erisman et al. (2011); 16 = Fourriere et al. (2016); 17 = Fourriere et al. (2017); 18 = Galván-Villa et al. (2016); 19 = González-Murcia et al. (2012); 20 = Graham (1975); 21 = Hooker (2009); 22 = López & Bussing (1982); 23 = Martínez-Muñoz et al. (2016); 24 = Murase et al. (2014); 25 = Palacios-Salgado et al. (2012); 26 = Palacios-Salgado et al. (2014); 27 = Salas et al. (2015); 28 = Shervette et al. (2007); 29 = Tavera & Rojas-Vélez (2017); 30 = Torres-Hernández et al. (2016); 31 = Tornabene et al. (2012); 32 = Van der Heiden & Findley (1988); 33 = Villareal-Cavazos et al. (2000). (DOCX) [file pone.0202863.s001.docx]

**S1 Checklist. Update checklist of gobies from Eastern Tropical Pacific with synonyms and records from literature.** Habitat, m= marine, e= estuarine, fw= freshwater, br= brackish. Source, *revised species from collected specimens or from museums and fish collections, **obtained from GBIF or literature. Literature: 1= Abbott (1989); 2= Alzate et al. (2012); 3= Alzate et al. (2014), 4= Béarez, P. (1996), 5= Béarez et al. (2007), 6= Castellanos‐Galindo et al. (2005); 7= Castellanos-Galindo & Krumme (2013); 8= Castellanos-Galindo et al. (2014); 9= Cortés (2012); 10= De la Cruz-Agüero et al. (1994); 11= Del Moral-Flores et al. (2013); 12= Del Moral-Flores et al. (2016); 13= Del Moral Flores et al. (2017); 14= Díaz-Ruiz et al. (2004); 15= Erisman et al. (2011); 16= Fourriere et al. (2016); 17= Fourriere et al. (2017); 18= Galván-Villa et al. (2016); 19= González-Murcia et al. (2012); 20= Graham (1975); 21= Hooker (2009); 22= López & Bussing (1982); 23= Martínez-Muñoz et al. (2016); 24= Murase et al. (2014); 25= Palacios-Salgado et al. (2012); 26= Palacios-Salgado et al. (2014); 27= Salas et al. (2015); 28= Shervette et al. (2007); 29= Tavera & Rojas-Vélez (2017); 30= Torres-Hernández et al. (2016); 31= Tornabene et al. (2012); 32= Van der Heiden & Findley (1988); 33= Villareal-Cavazos et al. (2000).

| **Family** | **Subfamily** | **Tribu** | **Group** | **Genus** | **Species** | **Synonyms** | **Habitat** | **Source** | **Literature** |
| --- | --- | --- | --- | --- | --- | --- | --- | --- | --- |
| Gobiidae Cuvier, 1816 | | | |  |  |  |  |  |  |
|  | Gobiinae Cuvier, 1816 | | |  |  |  |  |  |  |
|  |  |  |  |  |  |  |  |  |  |
|  |  |  |  | *Bathygobius* Bleeker, 1878 | | |  |  |  |
|  |  |  |  |  | *Bathygobius andrei* (Sauvage, 1880) | | m | * | 4, 7, 8, 22, 24, 30 |
|  |  |  |  |  |  | *Bathygobius andrei heteropoma* Ginsburg, 1947 |  |  |  |
|  |  |  |  |  | *Bathygobius lineatus* (Jenyns, 1842) | | m | * | 17, 28 |
|  |  |  |  |  |  | *Gobius lineatus* Jenyns, 1841 |  |  |  |
|  |  |  |  |  |  | *Gobius arundelii* Garman,1899 |  |  |  |
|  |  |  |  |  |  | *Bathygobius lineatus lupinus* Ginsburg, 1947 |  |  |  |
|  |  |  |  |  | *Bathygobius ramosus* Ginsburg, 1947 | | m | * | 4, 6, 8, 9, 10, 11, 12, 13, 15, 17, 19, 22, 25, 26, 27, 30, 32, 33 |
|  |  |  |  |  |  | *Gobius arundelii* Garman, 1899 |  |  |  |
|  |  |  |  |  |  | *Bathygobius ramosus curticeps* Ginsburg, 1947 |  |  |  |
|  |  |  |  |  |  | *Bathygobius ramosus longipinnis* Ginsburg, 1947 |  |  |  |
|  |  |  |  |  |  | *Bathygobius ramosus micromma* Ginsburg, 1947 |  |  |  |
|  |  |  |  |  |  | *Bathygobius ramosus ramosus* Ginsburg, 1947 |  |  |  |
|  |  |  |  | *Coryphopterus* Gill, 1863 | | |  |  |  |
|  |  |  |  |  | *Coryphopterus urospilus* Ginsburg, 1938 | | m | * | 3, 9, 10, 11, 12, 13, 15, 16, 17, 18, 21, 22, 25, 26, 27, 30, 32, 33 |
|  |  |  |  | *Evermannia* Jordan, 1895 | | |  |  |  |
|  |  |  |  |  | *Evermannia erici*Bussing, 1983 | | m | * |  |
|  |  |  |  |  | *Evermannia longipinnis* (Steindachner, 1879) | | m | ** | 11 |
|  |  |  |  |  |  | *Gobiosoma longipinne* Steindachner, 1879 |  |  |  |
|  |  |  |  |  | *Evermannia panamensis* Gilbert & Starks, 1904 | | m | * | 22,24 |
|  |  |  |  |  | *Evermannia zosterura* (Jordan & Gilbert, 1882) | | m | * | 4, 13, 22, 32 |
|  |  |  |  |  |  | *Gobiosoma zosterurum* Jordan & Gilbert, 1882 |  |  |  |
|  |  |  |  | *Lophiogobius* Günther, 1873 | | |  |  |  |
|  |  |  |  |  | *Lophogobius cristulatus* Ginsburg, 1939 | | m | * | 22, 24 |
|  |  |  |  |  | *Lophogobius cyprinoides* (Pallas, 1770) | | m, e, fw | ** |  |
|  |  |  |  |  |  | *Gobius cyprinoides* Pallas, 1770 |  |  |  |
|  |  |  |  |  |  | *Gobius cristagalli* Valenciennes, 1837 |  |  |  |
|  |  |  |  |  |  | *Lophogobius androsensis* Breder, 1932 |  |  |  |
|  |  |  |  | *Lythrypnus* Jordan & Evermann, 1896 | | |  |  |  |
|  |  |  |  |  | *Lythrypnus alphigena* Bussing, 1990 | | m | ** | 9, 16, 17 |
|  |  |  |  |  | *Lythrypnus cobalus* Bussing, 1990 | | m | * | 9, 16, 17 |
|  |  |  |  |  | *Lythrypnus dalli* (Gilbert, 1890) | | m | * | 5, 11, 16, 17, 21, 25, 32, 33 |
|  |  |  |  |  |  | *Gobius dalli* Gilbert, 1890 |  |  |  |
|  |  |  |  |  |  | *Lythrypnus crinitus* Ginsburg, 1939 |  |  |  |
|  |  |  |  |  |  | *Lythrypnus latifascia* Ginsburg, 1939 |  |  |  |
|  |  |  |  |  |  | *Microgobius cinctus* Nichols, 1952 |  |  |  |
|  |  |  |  |  | *Lythrypnus gilberti* (Heller & Snodgrass, 1903) | | m | * |  |
|  |  |  |  |  |  | *Gobius gilberti* Heller & Snodgrass, 1903 |  |  |  |
|  |  |  |  |  | *Lythrypnus insularis* Bussing, 1990 | | m | * | 12 |
|  |  |  |  |  | *Lythrypnus lavenbergi* Bussing, 1990 | | m | ** | 9, 16, 17 |
|  |  |  |  |  | *Lythrypnus pulchellus* Ginsburg, 1938 | | m | * | 11, 12, 15, 25, 32, 33 |
|  |  |  |  |  | *Lythrypnus rhizophora* (Heller & Snodgrass, 1903) | | m | * | 9, 16, 17, 22, 27 |
|  |  |  |  |  |  | *Gobius rhizophora* Heller & Snodgrass, 1903 |  |  |  |
|  |  |  |  |  | *Lythrypnus solanensis* Acero, 1981 | | m | ** |  |
|  |  |  |  |  | *Lythrypnus zebra* (Gilbert, 1890) | | m | ** | 12 |
|  |  |  |  |  |  | *Gobius zebra* Gilbert, 1890 |  |  |  |
|  |  | Gobiosomatini Birdsong, 1975 | | | | |  |  |  |
|  |  |  | Gobiosoma | | | |  |  |  |
|  |  |  |  | *Aboma* Jordan & Starks, 1895 | | |  |  |  |
|  |  |  |  |  | *Aboma etheostoma* Jordan & Starks, 1895 | | m, e | * | 13, 14, 22, 32, |
|  |  |  |  | *Aruma* Ginsburg, 1933 | | |  |  |  |
|  |  |  |  |  | *Aruma histrio* (Jordan, 1884) | | m, e | * | 25, 32, 33 |
|  |  |  |  |  |  | *Gobiosoma histrio J*ordan, 1884 |  |  |  |
|  |  |  |  |  |  | *Gobiosoma occidentale* Ginsburg, 1933 |  |  |  |
|  |  |  |  | *Barbulifer* Eigenmann & Eigenmann, 1888 | | |  |  |  |
|  |  |  |  |  | *Barbulifer ceuthoecus* (Jordan & Gilbert, 1884) | | m | * |  |
|  |  |  |  |  |  | *Gobiosoma ceuthoecum* Jordan & Gilbert, 1884 |  |  |  |
|  |  |  |  |  |  | *Barbulifer papillosus* Eigenmann & Eigenmann, 1888 |  |  |  |
|  |  |  |  |  | *Barbulifer mexicanus* Hoese & Larson, 1985 | | m | ** | 13, 15, 30, 33 |
|  |  |  |  |  | *Barbulifer pantherinus* (Pellegrin, 1901) | | m | * | 11, 25, 33 |
|  |  |  |  |  |  | *Gobiosoma pantherinum* Pellegrin, 1901 |  |  |  |
|  |  |  |  |  |  | *Gobiosoma spiritisancti* Wade, 1946 |  |  |  |
|  |  |  |  | *Chriolepis* Gilbert, 1892 | | |  |  |  |
|  |  |  |  |  | *Chriolepis atrimelum* Bussing, 1997 | | m | ** | 9, 16, 17 |
|  |  |  |  |  | *Chriolepis cuneata* Bussing, 1990 | | m | * | 2, 11, 13, 25, 27, 29 |
|  |  |  |  |  | *Chriolepis dialepta* Bussing, 1990 | | m | * | 9, 16, 17 |
|  |  |  |  |  | *Chriolepis lepidota* Findley, 1975 | | m | ** | 20 |
|  |  |  |  |  | *Chriolepis minutilla* Gilbert 1892 | | m | ** | 11 |
|  |  |  |  |  | *Chriolepis semisquamata* (Rutter, 1904) | | m | ** | 11, 25 |
|  |  |  |  |  |  | *Pycnomma semisquamatum* Rutter, 1904 |  |  |  |
|  |  |  |  |  | *Chriolepis tagus* Ginsburg, 1953 | | m | ** |  |
|  |  |  |  |  | *Chriolepis zebra* Ginsburg, 1938 | | m | * | 11, 25, 32, 33 |
|  |  |  |  | *Gobiosoma* Girard, 1858 | | |  |  |  |
|  |  |  |  |  | *Gobiosoma aceras* Ginsburg, 1939 | | m | * | 22 |
|  |  |  |  |  | *Gobiosoma chiquita* (Jenkins & Evermann, 1889) | | m, e | * | 11, 25, 33 |
|  |  |  |  |  |  | *Gobius chiquita* Jenkins & Evermann, 1889 |  |  |  |
|  |  |  |  |  | *Gobiosoma hildebrandi* (Ginsburg 1939) | | m, e | ** | 22 |
|  |  |  |  |  |  | *Garmannia hildebrandi* Ginsburg, 1939 |  |  |  |
|  |  |  |  |  | *Gobiosoma homochroma* (Ginsburg, 1939) | | m, e | ** | 22 |
|  |  |  |  |  |  | *Garmannia homochroma* Ginsburg, 1939 |  |  |  |
|  |  |  |  |  | *Gobiosoma nudum* (Meek & Hildebrand, 1928) | | m, br | * | 11, 22, 32, 33 |
|  |  |  |  |  |  | *Gerhardinus nudus* Meek & Hildebrand, 1928 |  |  |  |
|  |  |  |  |  | *Gobiosoma paradoxum* (Günther, 1861) | | m, br | * | 4, 11, 22, 26, 32 |
|  |  |  |  |  |  | *Gobius paradoxus* Günther, 1861 |  |  |  |
|  |  |  |  |  | *Gobiosoma seminudum* (Günther, 1861) | | m | * | 22, 25, 32 |
|  |  |  |  |  |  | *Gobius seminudus* Günther, 1861 |  |  |  |
|  |  |  |  | *Elacatinus* Jordan, 1904 | | |  |  |  |
|  |  |  |  |  | *Elacatinus puncticulatus* (Ginsburg, 1938) | | m | * | 3, 11, 13, 15, 18, 21, 22, 25, 26, 27, 30, 32, 33 |
|  |  |  |  |  |  | *Gobiosoma puncticulatum* Ginsburg, 1938 |  |  |  |
|  |  |  |  |  |  | *Gobiosoma rubrifrons* Fowler, 1944 |  |  |  |
|  |  |  |  | *Tigrigobius* Fowler, 1931 | | |  |  |  |
|  |  |  |  |  | *Tigrigobius digueti* (Pellegrin, 1901) | | m | * | 11, 15, 22, 32, 33 |
|  |  |  |  |  |  | *Gobiosoma brocki* Ginsburg, 1938 |  |  |  |
|  |  |  |  |  |  | *Gobiosoma digueti* Pellegrin, 1901 |  |  |  |
|  |  |  |  |  | *Tigrigobius inornatus* Bussing, 1990 | | m | * | 27 |
|  |  |  |  |  | *Tigrigobius janssi* Bussing, 1981 | | m | * | 22, 27 |
|  |  |  |  |  | *Tigrigobius limbaughi* Hoese & Reader, 2001 | | m | ** | 11, 15, 25 |
|  |  |  |  |  | *Tigrigobius nesiotes* Bussing, 1990 | | m | * | 9, 16, 17 |
|  |  |  |  | *Eleotrica* Ginsburg, 1933 | | |  |  |  |
|  |  |  |  |  | *Eleotrica cableae* Ginsburg, 1933 | | m | ** |  |
|  |  |  |  | *Gobulus* Ginsburg, 1933 | | |  |  |  |
|  |  |  |  |  | *Gobulus birdsongi* Hoese & Reader, 2001 | | m, e | ** |  |
|  |  |  |  |  | *Gobulus crescentalis* (Gilbert, 1892) | | m | * | 2, 3, 9, 11, 16, 17, 22, 27, 33 |
|  |  |  |  |  |  | *Gobiosoma crescentalis* Gilbert, 1892 |  |  |  |
|  |  |  |  |  | *Gobulus hancocki* Ginsburg, 1938 | | m | * | 8, 9, 11, 16, 17, 22, 27, 32 |
|  |  |  |  | *Gymneleotris* Bleeker, 1874 | | |  |  |  |
|  |  |  |  |  | *Gymneleotris seminuda* (Günther, 1864) | | m | * | 11, 15, 22, 27, 30, 32, 33 |
|  |  |  |  |  |  | *Eleotris seminudus* Günther, 1864 |  |  |  |
|  |  |  | Microgobius | | | |  |  |  |
|  |  |  |  | *Akko* Birdsong & Robins, 1995 | | |  |  |  |
|  |  |  |  |  | *Akko brevis* (Günther, 1864) | | m | * |  |
|  |  |  |  |  |  | *Amblyopus brevis* Günther, 1864 |  |  |  |
|  |  |  |  |  | *Akko rossi* Van Tassell & Baldwin 2004 | | m | ** |  |
|  |  |  |  | *Bollmannia* Jordan, 1890 | | |  |  |  |
|  |  |  |  |  | *Bollmannia chlamydes* Jordan, 1890 | | m | * | 4 |
|  |  |  |  |  | *Bollmannia macropoma* Gilbert, 1892 | | m | * | 11 |
|  |  |  |  |  | *Bollmannia marginalis* Ginsburg, 1939 | | m | * | 13, 30 |
|  |  |  |  |  | *Bollmannia ocellata* Gilbert, 1892 | | m | * | 11, 22, 23, |
|  |  |  |  |  |  | *Bollmania pawneea* Ginsburg, 1939 |  |  |  |
|  |  |  |  |  |  | *Bollmannia longipinnis* Ginsburg, 1939 |  |  |  |
|  |  |  |  |  | *Bollmannia stigmatura* Gilbert, 1892 | | m | ** | 13, 22, 23, 24, 30, |
|  |  |  |  |  | *Bollmannia umbrosa* Ginsburg, 1939 | | m | * | 13, 22, 24, 27, |
|  |  |  |  | *Microgobius* Poey, 1876 | | |  |  |  |
|  |  |  |  |  | *Microgobius brevispinis* Ginsburg, 1939 | | m, e | ** | 15, 22, 32 |
|  |  |  |  |  | *Microgobius crocatus* Birdsong, 1968 | | m, e | ** | 22, 24 |
|  |  |  |  |  | *Microgobius curtus* Ginsburg, 1939 | | m, e | ** | 22, 24 |
|  |  |  |  |  | *Microgobius cyclolepis* Gilbert, 1890 | | m, e | ** | 32 |
|  |  |  |  |  | *Microgobius emblematicus* (Jordan & Gilbert, 1882) | | m, e | ** | 4, 11, 22, 32 |
|  |  |  |  |  |  | *Gobius emblematicus* Jordan & Gilbert, 1882 |  |  |  |
|  |  |  |  |  | *Microgobius erectus* Ginsburg, 1938 | | m | ** | 13, 22, 24 |
|  |  |  |  |  | *Microgobius miraflorensis* Gilbert & Starks, 1904 | | m, e | ** | 4, 13, 22, 24, 26, 30, 32 |
|  |  |  |  |  | *Microgobius tabogensis* Meek & Hildebrand, 1928 | | m, e | ** | 4, 13, 22, 24, 28, 32 |
|  |  |  |  |  | *Microgobius urraca* Tornabene, van Tassell & Robertson 2012 | | m | ** | 31 |
|  |  |  |  | *Parrella* Ginsburg, 1938 | | |  |  |  |
|  |  |  |  |  | *Parrella fusca* Ginsburg, 1939 | | m | ** | 22 |
|  |  |  |  |  | *Parrella ginsburgi* Wade, 1946 | | m | ** | 22, 32 |
|  |  |  |  |  | *Parrella lucretiae* (Eigenmann & Eigenmann, 1888) | | m | ** | 13, 22, 24 |
|  |  |  |  |  |  | *Gobius lucretiae* Eigenmann & Eigenmann, 1888 |  |  |  |
|  |  |  |  |  |  | *Parrella spilopteryx* Ginsburg, 1939 |  |  |  |
|  |  |  |  |  | *Parrella maxillaris* Ginsburg, 1938 | | m | ** | 11, 22, 32 |
|  | Gobionellinae Bleeker, 1874 | | | | | |  |  |  |
|  |  |  |  | *Awaous* Valenciennes, 1837 | | |  |  |  |
|  |  |  |  |  | **?***Awaous transandeanus* (Günther, 1861) | | m, e, fw | ** |  |
|  |  |  |  |  |  | *Gobius banana* Valenciennes, 1837 |  |  |  |
|  |  |  |  |  |  | *Awaous nelsoni* Evermann, 1898 |  |  |  |
|  |  |  |  |  |  | *Gobius transandeanus* Günther, 1861 |  |  |  |
|  |  |  |  | *Ctenogobius* Gill, 1858 | | |  |  |  |
|  |  |  |  |  | *Ctenogobius manglicola* (Jordan & Starks in Jordan, 1895) | | m, e | * | 4, 22, 24, 32 |
|  |  |  |  |  |  | *Gobius manglicola* Jordan & Starks, 1895 |  |  |  |
|  |  |  |  |  | *Ctenogobius sagittula* (Günther, 1862) | | m, e | * | 4, 7, 10, 11, 13, 14, 18, 24, 25, 28, 30, 32 |
|  |  |  |  |  |  | *Gobius longicaudus* Jenkins & Evermann, 1889 |  |  |  |
|  |  |  |  |  |  | *Euctenogobius sagittula* Günther, 1862 |  |  |  |
|  |  |  |  | *Evorthodus* Gill, 1859 | | |  |  |  |
|  |  |  |  |  | *Evorthodus minutus* Meek & Hildebrand, 1928 | | m, e | * | 4, 22, 24, 28 |
|  |  |  |  | *Gillichthys* Cooper, 1864 | | |  |  |  |
|  |  |  |  |  | *Gillichthys detrusus* Gilbert & Scofield, 1898 | | m, e, fw | ** |  |
|  |  |  |  |  | *Gillichthys mirabilis* Cooper, 1864 | | m, e, fw | * | 11 |
|  |  |  |  |  |  | *Aprolepis barbarae* Hubbs, 1921 |  |  |  |
|  |  |  |  |  |  | *Gobius townsendi* Eigenmann & Eigenmann, 1889 |  |  |  |
|  |  |  |  |  | *Gillichthys seta* (Ginsburg, 1938) | | m | * | 11 |
|  |  |  |  |  |  | *Lepidogobius seta* Ginsburg, 1938 |  |  |  |
|  |  |  |  | *Gobioides* Lacepède, 1800 | | |  |  |  |
|  |  |  |  |  | *Gobioides peruanus* (Steindachner, 1880) | | e, fw | * | 1, 4, 24 |
|  |  |  |  |  |  | *Amblyopus peruanus* Steindachner, 1880 |  |  |  |
|  |  |  |  | *Gobionellus* Girard, 1858 | | |  |  |  |
|  |  |  |  |  | *Gobionellus daguae* (Eigenmann, 1918) | | m, e | ** | 22 |
|  |  |  |  |  |  | *Gobius daguae* Eigenmann, 1918 |  |  |  |
|  |  |  |  |  |  | *Euctenogobius panamensis* Meek & Hildebrand, 1928 |  |  |  |
|  |  |  |  |  | *Gobionellus liolepis* (Meek & Hildebrand, 1928) | | m | * | 22, 24, 28 |
|  |  |  |  |  |  | *Euctenogobius liolepis* Meek & Hildebrand, 1928 |  |  |  |
|  |  |  |  |  | *Gobionellus microdon* (Gilbert, 1892) | | m, e, fw | * | 4, 13, 14, 22, 28, 30, 32 |
|  |  |  |  |  |  | *Gobius microdon* Gilbert, 1892 |  |  |  |
|  |  |  |  |  |  | *Gobionellus mystax* Ginsburg, 1953 |  |  |  |
|  |  |  |  | *Ilypnus* Jordan & Evermann 1896 | | |  |  |  |
|  |  |  |  |  | *Ilypnus gilberti* (Eigenmann & Eigenmann, 1889) | | m | * | 10, 11 |
|  |  |  |  |  |  | *Lepidogobius gilberti* Eigenmann & Eigenmann, 1889 |  |  |  |
|  |  |  |  |  | *Ilypnus luculentus* (Ginsburg, 1938) | | m | ** | 11 |
|  |  |  |  |  |  | *Lepidogobius luculentus* Ginsburg, 1938 |  |  |  |
|  |  |  |  | *Quietula* Jordan and Evermann, 1895 | | |  |  |  |
|  |  |  |  |  | *Quietula guaymasiae* (Jenkins & Evermann, 1889) | | m, e | ** | 11 |
|  |  |  |  |  |  | *Gillichthys guaymasiae* Jenkins & Evermann, 1889 |  |  |  |
|  |  |  |  |  | *Quietula y-cauda* (Jenkins & Evermann, 1889) | | m, e | ** | 11, 25 |
|  |  |  |  |  |  | *Gillichthys guaymasiae* Jenkins & Evermann, 1889 |  |  |  |
|  |  |  |  |  |  | *Gillichthys y-cauda* Jenkins & Evermann, 1889 |  |  |  |

**References**

Abbott, J. F. (1899). The marine fishes of Peru. *Proceedings of the Academy of Natural Sciences of Philadelphia*, *51*, 324-364

Alzate, A., Muñoz, C. G., Zapata, F. A., & Giraldo, A. (2012). New records of cryptobenthic fishes in coral reef habitats of Gorgona Island, Colombia, Tropical Eastern Pacific. *Boletín de Investigaciones Marinas y Costeras-INVEMAR*, *41*, 229-235. https://doi.org/10.25268/bimc.invemar.2012.41.1.82

Alzate, A., Zapata, F. A., & Giraldo, A. (2014). A comparison of visual and collection-based methods for assessing community structure of coral reef fishes in the Tropical Eastern Pacific. *Revista de Biología Tropical*, *62*, 359-371. https://doi.org/10.15517/rbt.v62i0.16361

Bearez, P. (1996). Lista de los peces marinos del Ecuador continental. *Revista de Biología Tropical*, 44, 731-741.

Béarez, P., Bujard, J. T., & Campoverde, R. (2007). Description of four small reef fishes from Ecuador: *Oxycirrhites typus* (Cirrhitidae), *Acanthemblemaria balanorum* (Chaenopsidae), *Arcos decoris* (Gobiesocidae) and *Lythrypnus dalli* (Gobiidae). *Cybium*, 31, 477-479.

Castellanos‐Galindo, G. A., Giraldo, A., & Rubio, E. A. (2005). Community structure of an assemblage of tidepool fishes on a tropical eastern Pacific rocky shore, Colombia. *Journal of Fish Biology, 67*, 392-408. https://doi.org/10.1111/j.0022-1112.2005.00735.x.

Castellanos-Galindo, G. A., & Krumme, U. (2013). Tidal, diel and seasonal effects on intertidal mangrove fish in a high-rainfall area of the Tropical Eastern Pacific. *Marine Ecology Progress Series*, *494*, 249-265. https://doi.org/10.3354/meps10512.

Castellanos-Galindo, G. A., Giraldo, A., & Zapata, F. A. (2014). Tidepool fish assemblages of Gorgona Island, Colombian Pacific coast: a local and regional comparison. *Revista de Biología Tropical*, *62*, 373-390. https://doi.org/10.15517/rbt.v62i0.16362.

Cortés, J. (2012). Marine biodiversity of an Eastern Tropical Pacific oceanic island, Isla del Coco, Costa Rica. *Revista de Biología Tropical*, *60*, 131-185.

De la Cruz-Agüero, J. L., Galvan-Magana, F., Abitia-Cardenas, L. A., Rodriguez-Romero, J., & Gutierrez-Sanchez, F. J. (1994). Systematic list of marine fishes from Bahia Magdalena, Baja California Sur (Mexico). *Ciencias marinas, 20*, 17-31.

Del Moral-Flores, L. F., González-Acosta, A. F., Espinosa-Pérez, H., Ruiz-Campos, G., & Castro-Aguirre, J. L. (2013). Lista anotada de la ictiofauna de las islas del golfo de California, con comentarios sobre sus afinidades zoogeográficas. *Revista mexicana de biodiversidad, 84*, 184-214. http://dx.doi.org/10.7550/rmb.27335

Del Moral-Flores, L. F., Gracián-Negrete, J. M., & Guzmán-Camacho, A. F. (2016). Peces del archipiélago de las islas Revillagigedo: una actualización sistemática y biogeográfica. *Biocyt, 9*, 596-619.

Del Moral-Flores, L. F., Anislado-Tolentino, V., Martínez-Ramírez, E., de León, G. P. P., Ramírez-Antonio, E., & González-Medina, G. (2017). Ictiofauna marina de Oaxaca, México: listado sistemático y afinidades zoogeográficas. *Acta Universitaria, 27*, 3-25. https://doi.org/10.15174/au.2016.1069.

Díaz-Ruiz, S., Cano-Quiroga, E., Aguirre-León, A., & Ortega-Bernal, R. (2004). Diversidad, abundancia y conjuntos ictiofaunísticos del sistema lagunar-estuarino Chantuto-Panzacola, Chiapas, México. *Revista de Biología Tropical*, *52*, 187-199. https://doi.org/10.15517/rbt.v52i1.14879.

Erisman, B. E., Galland, G. R., Mascarenas, I., Moxley, J., Walker, H. J., Aburto-Oropeza, O., ... & Ezcurra, E. (2011). List of coastal fishes of Islas Marías archipelago, Mexico, with comments on taxonomic composition, biogeography, and abundance. *Zootaxa, 2985*, 26-40.

Fourriére, M., Reyes-Bonilla, H., Ayala-Bocos, A., Ketchum, J. A., & Chávez-Comparan, J. C. (2016). Checklist and analysis of completeness of the reef fish fauna of the Revillagigedo Archipelago, Mexico. *Zootaxa, 4150*, 436-466. https://doi.org/10.11646/zootaxa.4150.4.4.

Fourriére, M., Alvarado, J. J., Bocos, A. A., & Cortés, J. (2017). Updated checklist and analysis of completeness of the marine fish fauna of Isla del Coco, Pacific of Costa Rica. *Marine Biodiversity, 47*, 813-821. https://doi.org/10.1007/s12526-016-0501-6.

Galván-Villa, C. M., Ríos-Jara, E., Bastida-Izaguirre, D., Hastings, P. A., & Balart, E. F. (2016). Annotated checklist of marine fishes from the Sanctuary of Bahía Chamela, Mexico with occurrence and biogeographic data. *Zookeys, 554*, 139. https://doi.org/10.3897/zookeys.554.6106.

González-Murcia, S., Marín-Martínez, C., & Ayala-Bocos, A. (2012). Intertidal rockpool icthyofauna of El Pital, La Libertad, El Salvador. *Check List, 8*, 1216-1220. https://doi.org/10.15560/8.6.1216.

Graham J. B. 1975. The Biological Investigation of Malpelo Island, Colombia*. Smithsonian Contribution to Zoology, 176*, 1-98. https://doi.org/10.5479/si.00810282.176.

Hooker, M. (2009). Nuevos registros de peces costeros tropicales para el Perú. *Revista Peruana de Biología, 16*, 33-41. https://doi.org/10.15381/rpb.v16i1.192.

López, M. I., & Bussing, W. A. (1982). Lista provisional de los peces marinos de la costa Pacífica de Costa Rica. *Revista de Biologia Tropical*, *30*, 5-26.

Martínez-Muñoz, M. A., Lloris, D., Gracia, A., Ramírez-Murillo, R., Sarmiento-Nafáte, S., Ramos-Cruz, S., & Fernández, F. (2016). Biogeographical affinities of fish associated to the shrimp trawl fishery in the Gulf of Tehuantepec, Mexico. *Revista de Biología Tropical*, *64*, 683-700. https://doi.org/10.15517/rbt.v64i2.19034.

Murase, A., Angulo, A., Miyazaki, Y., Bussing, W., & López, M. (2014). Marine and estuarine fish diversity in the inner Gulf of Nicoya, Pacific coast of Costa Rica, Central America. *Check List, 10*, 1401. https://doi.org/10.15560/10.6.1401.

Palacios-Salgado, D. S., Moreno-Sanchez, X. G., Abitia-Cardenas, L. A., Gutierrez-Sanchez, F. J., & Rodriguez-Romero, J. (2012). Ichthyodiversity of San Jose, San Francisquito, and El Pardito Islands in the Southwestern Gulf of California, Mexico. *Acta Ichthyologica Et Piscatoria*, *42*, 177-191. https://doi.org/10.3750/aip2011.42.3.03.

Palacios-Salgado, D. S., Ramírez-Valdez, A., Rojas-Herrera, A. A., Amores, J. G., & Melo-García, M. A. (2014). Marine fishes of Acapulco, Mexico (Eastern Pacific Ocean*). Marine Biodiversity, 44*, 471–490. https://doi.org/10.1007/s12526-014-0209-4.

Salas, E., Sánchez-Godínez, C., & Montero-Cordero, A. (2015). Peces marinos de la Reserva Biológica Isla del Caño: Estructura de las comunidades de peces de arrecife y lista taxonómica actualizada de los peces costeros. *Revista de Biología Tropical*, *63*, 97-116.

Shervette, V. R., Aguirre, W. E., Blacio, E., Cevallos, R., Gonzalez, M., Pozo, F., & Gelwick, F. (2007). Fish communities of a disturbed mangrove wetland and an adjacent tidal river in Palmar, Ecuador. *Estuarine, Coastal and Shelf Science*, *72*, 115-128. https://doi.org/10.1016/j.ecss.2006.10.010.

Tavera, J., & Rojas-Vélez, S. (2017). Seeing the invisible: *Chriolepis lepidota* (Gobiidae), literally as never seen before. *Marine Biodiversity Records*, 10, 23. https://doi.org/10.1186/s41200-017-0125-y.

Torres-Hernández, E., Palacios-Morales, G., Romero-Gallardo, S., Salazar-Araujo, P., García-Meraz, A., Madrigal-Guridi, X., ... & Domínguez-Domínguez, O. (2016). Annotated checklist of the coastal ichthyofauna from Michoacán State, Mexico. *ZooKeys*, *606*, 99-126. https://doi.org/10.3897/zookeys.606.9004.

Tornabene, L., Van Tassell, J. L., & Robertson, D. R. (2012). *Microgobius urraca* (Teleostei: Gobiidae), a new species of goby from the tropical eastern Pacific. *Zootaxa*, 3447, 41-55. https://doi.org/10.11646/zootaxa.462.1.1.

Van der Heiden, A. M., & Findley, L. T. (1988). Lista de los peces marinos del sur de Sinaloa, México. *Anales del Instituto de Ciencias del Mar y Limnología*, *15*, 209-224.

Villarreal-Cavazos, A., Reyes-Bonilla, H., Bermúdez-Almada, B., & Arizpe-Covarrubias, O. (2000). Los peces del arrecife de Cabo Pulmo, Golfo de California, México: Lista sistemática y aspectos de abundancia y biogeografía. *Revista de Biología Tropical*, *48*, 413-424.
