# Supplementary material for: Gamma-diversity partitioning of gobiid fishes (Teleostei: Gobiidae) ensemble along of Eastern Tropical Pacific: Biological inventory, latitudinal variation and species turnover
Source: PLoS One. 2018 Aug 31;13(8):e0202863. doi: 10.1371/journal.pone.0202863 (PMC6118385; doi:10.1371/journal.pone.0202863)
Supplement: S1 Fig — Ecoregions abbreviations are in Fig 1. Provinces: Cold Temperate Northeast Pacific (CTNP), Warm Temperate Northeast Pacific (WTNP), Tropical East Pacific (TEaP), Galapagos (Gala). Realms: Temperate Northern Pacific (TNP), Eastern Tropical Pacific (ETP). (DOCX) [file pone.0202863.s003.docx]

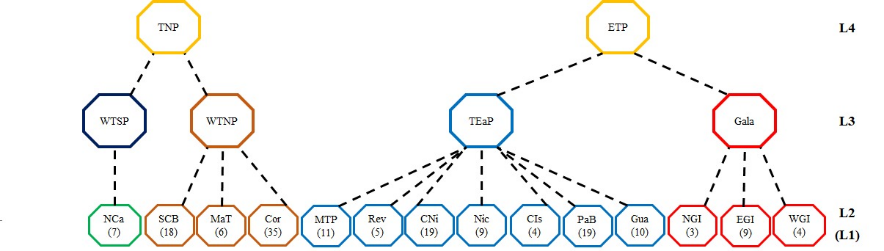


**S1 Fig. Following to Spalding et al. (2007), gobies distribution was grouped in 159 sampling units (First level, L1), 14 ecoregions (Second level, L2), 4 provinces (Third level, L3) and 2 realms (Fourth level, L4).** **Ecoregions**: Northern California= NCa, Southern California Bight= SCB, Magdalena Transition= MaT, Cortezian= Cor, 5= Revillagigedos= Rev, Mexican Tropical Pacific= MTP, Chiapas-Nicaragua= CNi, Nicoya= Nic, Coco Island= CIs, Panama Bight= PaB, Guayaquil= Gua, Northern Galapagos Islands= NGI, Western Galapagos Islands= WGI, Eastern Galapagos Islands= EGI. **Provinces**: Cold Temperate Northeast Pacific= CTNP, Warm Temperate Northeast Pacific= WTNP, Tropical East Pacific= TEaP, Galapagos= Gala. **Realms**: Temperate Northern Pacific= TNP, Eastern Tropical Pacific= ETP.
