# Supplementary material for: Gamma-diversity partitioning of gobiid fishes (Teleostei: Gobiidae) ensemble along of Eastern Tropical Pacific: Biological inventory, latitudinal variation and species turnover
Source: PLoS One. 2018 Aug 31;13(8):e0202863. doi: 10.1371/journal.pone.0202863 (PMC6118385; doi:10.1371/journal.pone.0202863)
Supplement: S2 Fig — (DOCX) [file pone.0202863.s004.docx]

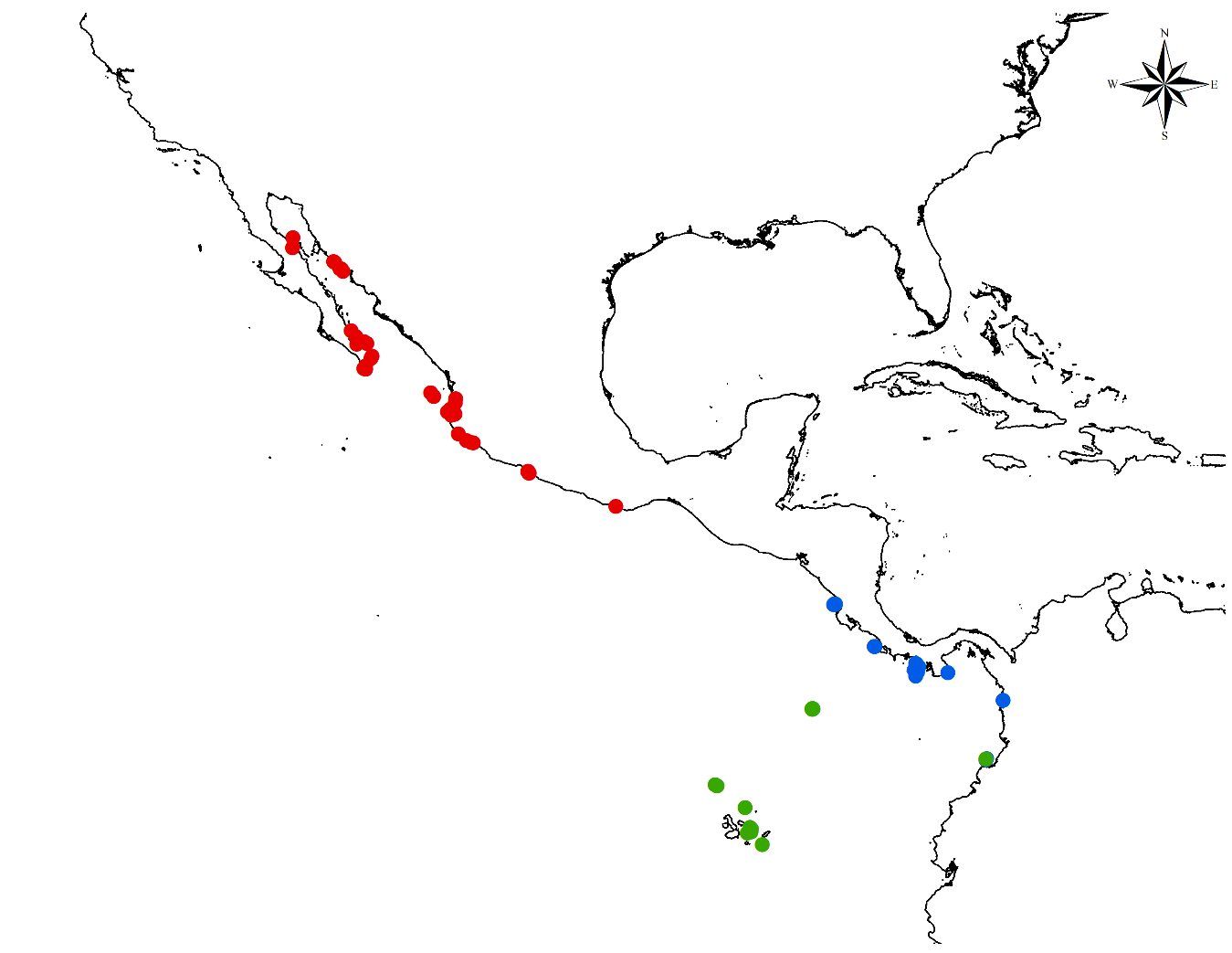


**S2 Fig. Distribution of *Tigrigobius digueti* (red), *Tigrigobius inornatus* (blue) and *Tigrigobius nesiotes* (green) in Eastern Tropical Pacific.**
