# Supplementary material for: Gamma-diversity partitioning of gobiid fishes (Teleostei: Gobiidae) ensemble along of Eastern Tropical Pacific: Biological inventory, latitudinal variation and species turnover
Source: PLoS One. 2018 Aug 31;13(8):e0202863. doi: 10.1371/journal.pone.0202863 (PMC6118385; doi:10.1371/journal.pone.0202863)
Supplement: S1 Table — Ecoregions: 1 = Northern California (NCa), 2 = Southern California Bight (SCB), 3 = Magdalena Transition (MaT), 4 = Cortezian (Cor), 5 = Revillagigedos (Rev), 6 = Mexican Tropical Pacific (MTP), 7 = Chiapas-Nicaragua (CNi), 8 = Nicoya (Nic), 9 = Coco Island (CIs), 10 = Panama Bight (PaB),11 = Guayaquil (Gua), 12 = Northern Galapagos Islands (NGI), 13 = Western Galapagos Islands (WGI), 14 = Eastern Galapagos Islands (EGI). Provinces: Cold Temperate Northeast Pacific (CTNP), Warm Temperate Northeast Pacific (WTNP), Tropical East Pacific (TEaP), Galapagos (Gala). Realms: Temperate Northern Pacific (TNP) and Eastern Tropical Pacific (ETP). (DOCX) [file pone.0202863.s005.docx]

**S1 Table. Number of genera and species in each ecoregion, province and realms. Ecoregions**: 1= Northern California (NCa), 2= Southern California Bight (SCB), 3= Magdalena Transition (MaT), 4= Cortezian (Cor), 5= Revillagigedos (Rev), 6= Mexican Tropical Pacific (MTP), 7= Chiapas-Nicaragua (CNi), 8= Nicoya (Nic), 9= Coco Island (CIs), 10= Panama Bight (PaB),11= Guayaquil (Gua), 12= Northern Galapagos Islands (NGI), 13= Western Galapagos Islands (WGI), 14= Eastern Galapagos Islands (EGI). **Provinces**: Cold Temperate Northeast Pacific (CTNP), Warm Temperate Northeast Pacific (WTNP), Tropical East Pacific (TEaP), Galapagos (Gala). **Realms**: Temperate Northern Pacific (TNP) and Eastern Tropical Pacific (ETP).

| **Ecoregion** | **Genera** | **Species** | **Provinces** | **Genera** | **Species** | **Realms** | **Genera** | **Species** |
| --- | --- | --- | --- | --- | --- | --- | --- | --- |
| NCa | 6 | 7 | CTNP | 6 | 7 | ETP | 23 | 73 |
| SCB | 9 | 10 | WTNP | 23 | 51 | TNP | 23 | 51 |
| MaT | 12 | 18 | TEaP | 22 | 71 |  |  |  |
| Cor | 22 | 50 | Gala | 7 | 9 |  |  |  |
| Rev | 3 | 6 |  |  |  |  |  |  |
| MTP | 15 | 21 |  |  |  |  |  |  |
| CNi | 19 | 38 |  |  |  |  |  |  |
| Nic | 18 | 37 |  |  |  |  |  |  |
| CIs | 5 | 10 |  |  |  |  |  |  |
| PaB | 21 | 54 |  |  |  |  |  |  |
| Gua | 14 | 26 |  |  |  |  |  |  |
| NGI | 4 | 5 |  |  |  |  |  |  |
| WGI | 5 | 6 |  |  |  |  |  |  |
| EGI | 6 | 8 |  |  |  |  |  |  |
