# Supplementary material for: Gamma-diversity partitioning of gobiid fishes (Teleostei: Gobiidae) ensemble along of Eastern Tropical Pacific: Biological inventory, latitudinal variation and species turnover
Source: PLoS One. 2018 Aug 31;13(8):e0202863. doi: 10.1371/journal.pone.0202863 (PMC6118385; doi:10.1371/journal.pone.0202863)
Supplement: S2 Table — (DOCX) [file pone.0202863.s006.docx]

**S2 Table.** Beta diversity partitioning outputs of species turnover and nestedness components among ecoregions.

|  | **Pairwise**  **comparisons** | **Turnover** | **Nestedness** | **Average turnover** | **Average nestedness** |
| --- | --- | --- | --- | --- | --- |
| **Global test** |  | 0.813 | 0.115 |  |  |
|  |  |  |  |  |  |
| **Coastal ecoregions** | NCa-SCB | 0.500 | 0.136 | 0.274 | 0.297 |
|  | SCB-MaT | 0.364 | 0.303 |  |  |
|  | MaT-Cor | 0.100 | 0.547 |  |  |
|  | Cor-MTP | 0.100 | 0.547 |  |  |
|  | MTP-CNi | 0.417 | 0.258 |  |  |
|  | CNi-Nic | 0.311 | 0.043 |  |  |
|  | Nic-PaB | 0.327 | 0.105 |  |  |
|  | PaB-Gua | 0.074 | 0.436 |  |  |
|  |  |  |  |  |  |
| **Revillagigedo *vs.* all ecoregions** | Rev-NCa | 0.800 | 0.000 | 0.715 | 0.159 |
|  | Rev-SCB | 0.800 | 0.046 |  |  |
|  | Rev-MaT | 0.500 | 0.310 |  |  |
|  | Rev-Cor | 0.500 | 0.423 |  |  |
|  | Rev-MTP | 0.667 | 0.197 |  |  |
|  | Rev-CNi | 0.500 | 0.400 |  |  |
|  | Rev-Nic | 0.667 | 0.265 |  |  |
|  | Rev-PaB | 0.800 | 0.163 |  |  |
|  | Rev-Gua | 0.800 | 0.133 |  |  |
|  | Rev-CIs | 0.800 | 0.057 |  |  |
|  | Rev-NGI | 0.909 | 0.000 |  |  |
|  | Rev-WGI | 0.750 | 0.028 |  |  |
|  | Rev-EGI | 0.800 | 0.046 |  |  |
|  |  |  |  |  |  |
| **Cocos Island *vs.* all ecoregions** | CIs-NCa | 0.909 | 0.024 | 0.866 | 0.056 |
|  | CIs-SCB | 1.000 | 0.000 |  |  |
|  | CIs-MaT | 0.947 | 0.017 |  |  |
|  | CIs-Cor | 0.889 | 0.077 |  |  |
|  | CIs-MTP | 0.947 | 0.017 |  |  |
|  | CIs-CNi | 0.824 | 0.110 |  |  |
|  | CIs-Nic | 0.889 | 0.070 |  |  |
|  | CIs-PaB | 0.824 | 0.124 |  |  |
|  | CIs-Gua | 0.947 | 0.024 |  |  |
|  | CIs-NGI | 0.667 | 0.103 |  |  |
|  | CIs-WGI | 0.750 | 0.096 |  |  |
|  | CIs-EGI | 0.800 | 0.013 |  |  |
|  |  |  |  |  |  |
| **Northern Galapagos Island *vs.* all ecoregions** | NGI-NCa | 1.000 | 0.000 | 0.841 | 0.053 |
|  | NGI-SCB | 1.000 | 0.000 |  |  |
|  | NGI-MaT | 1.000 | 0.000 |  |  |
|  | NGI-Cor | 1.000 | 0.000 |  |  |
|  | NGI-MTP | 1.000 | 0.000 |  |  |
|  | NGI-CNi | 0.909 | 0.068 |  |  |
|  | NGI-Nic | 0.909 | 0.069 |  |  |
|  | NGI-PaB | 0.909 | 0.073 |  |  |
|  | NGI-Gua | 0.909 | 0.059 |  |  |
|  | NGI-WGI | 0.333 | 0.095 |  |  |
|  | NGI-EGI | 0.286 | 0.214 |  |  |
|  |  |  |  |  |  |
| **Western Galapagos Island *vs.* all ecoregions** | WGI-NCa | 1.000 | 0.000 | 0.756 | 0.151 |
|  | WGI-SCB | 0.889 | 0.034 |  |  |
|  | WGI-MaT | 0.889 | 0.068 |  |  |
|  | WGI-Cor | 0.889 | 0.093 |  |  |
|  | WGI-MTP | 0.889 | 0.068 |  |  |
|  | WGI-CNi | 0.750 | 0.201 |  |  |
|  | WGI-Nic | 0.750 | 0.205 |  |  |
|  | WGI-PaB | 0.750 | 0.212 |  |  |
|  | WGI-Gua | 0.750 | 0.181 |  |  |
|  | WGI-EGI | 0.000 | 0.444 |  |  |
|  |  |  |  |  |  |
| **Eastern Galapagos Island *vs*. all ecoregions** | EGI-NCa | 0.909 | 0.019 | 0.795 | 0.119 |
|  | EGI-SCB | 0.875 | 0.000 |  |  |
|  | EGI-MaT | 0.875 | 0.048 |  |  |
|  | EGI-Cor | 0.800 | 0.146 |  |  |
|  | EGI-MTP | 0.941 | 0.022 |  |  |
|  | EGI-CNi | 0.714 | 0.193 |  |  |
|  | EGI-Nic | 0.615 | 0.274 |  |  |
|  | EGI-PaB | 0.714 | 0.213 |  |  |
|  | EGI-Gua | 0.714 | 0.157 |  |  |
