# Supplementary material for: Gamma-diversity partitioning of gobiid fishes (Teleostei: Gobiidae) ensemble along of Eastern Tropical Pacific: Biological inventory, latitudinal variation and species turnover
Source: PLoS One. 2018 Aug 31;13(8):e0202863. doi: 10.1371/journal.pone.0202863 (PMC6118385; doi:10.1371/journal.pone.0202863)
Supplement: S3 Table — (DOCX) [file pone.0202863.s007.docx]

**S3 Table.** Unique (found in a single sample) and duplicate species (found in two samples) were considered as unshared species (UD). Shared species (S) were species found in three or more samples (S). This table include the ecoregions and provinces were these species are shared or unshared. Acronyms are showed in Table A.

| **Species** | **UD** | **S** | **Ecoregions** | **UD** | **S** | **Provinces** |
| --- | --- | --- | --- | --- | --- | --- |
| *Aboma etheostoma* Jordan & Starks, 1895 |  | 4 | Cor, CNi, Nic, PaB | 2 |  | WTNP, TEaP |
| *Akko brevis* (Günther, 1864) |  | 3 | CNi, PaB, Gua | 1 |  | TEaP |
| *Akko rossi* Van Tassell & Baldwin 2004 | 1 |  | CNi | 1 |  | TEaP |
| *Aruma histrio* (Jordan, 1884) | 1 |  | Cor | 1 |  | WTNP |
| *Barbulifer ceuthoecus* (Jordan & Gilbert, 1884) | 1 |  | PaB | 1 |  | TEaP |
| *Barbulifer mexicanus* Hoese & Larson, 1985 |  | 3 | MaT, Cor, MTP | 2 |  | WTNP, TEaP |
| *Barbulifer pantherinus* (Pellegrin, 1901) | 1 |  | Cor | 1 |  | WTNP |
| *Bathygobius andrei* (Sauvage, 1880) |  | 5 | MTP, CNi, Nic, PaB, Gua | 1 |  | TEaP |
| *Bathygobius lineatus* (Jenyns, 1842) |  | 6 | Nic, PaB, Gua, NGI, EGI, WGI | 2 |  | TEaP, Gal |
| *Bathygobius ramosus* Ginsburg, 1947 |  | 11 | NCa, SCB, MaT, Cor, MTP, Rev, CNi, Nic, CIs, PaB, Gua |  | 3 | CTNP, WTNP, TEaP |
| *Bollmannia chlamydes* Jordan, 1890 |  | 5 | Cor, CNi, Nic, PaB, Gua | 2 |  | WTNP, TEaP |
| *Bollmannia macropoma* Gilbert, 1892 |  | 4 | Cor, CNi, Nic, PaB | 2 |  | WTNP, TEaP |
| *Bollmannia marginalis* Ginsburg, 1939 |  | 5 | Cor, MTP, Nic, PaB, Gua | 2 |  | WTNP, TEaP |
| *Bollmannia ocellata* Gilbert, 1892 |  | 5 | Cor, MTP, CNi, Nic, PaB, Gua | 2 |  | WTNP, TEaP |
| *Bollmannia stigmatura* Gilbert, 1892 |  | 5 | Cor, MTP, Nic, PaB, Gua | 2 |  | WTNP, TEaP |
| *Bollmannia umbrosa* Ginsburg, 1939 |  | 6 | Cor, MTP, CNi, Nic, PaB, Gua | 2 |  | WTNP, TEaP |
| *Chriolepis atrimelum* Bussing, 1997 | 1 |  | CIs | 1 |  | TEaP |
| *Chriolepis cuneata* Bussing, 1990 |  | 3 | Cor, CNi, Nic | 2 |  | WTNP, TEaP |
| *Chriolepis dialepta* Bussing, 1990 | 1 |  | CIs | 1 |  | TEaP |
| *Chriolepis lepidota* Findley, 1975 | 1 |  | PaB | 1 |  | TEaP |
| *Chriolepis minutilla* Gilbert 1892 | 1 |  | Cor | 1 |  | WTNP |
| *Chriolepis semisquamata* (Rutter, 1904) | 1 |  | Cor | 1 |  | WTNP |
| *Chriolepis tagus* Ginsburg, 1953 | 1 |  | WGI | 1 |  | Gal |
| *Chriolepis zebra* Ginsburg, 1938 | 2 |  | Cor, MTP | 2 |  | WTNP, TEaP |
| *Coryphopterus urospilus* Ginsburg, 1938 |  | 11 | SCB, MaT, Cor, MTP, Rev, CNi, Nic, PaB, Gua, EGI, WGI |  | 3 | WTNP, TEaP, Gal |
| *Ctenogobius manglicola* (Jordan & Starks in Jordan, 1895) |  | 5 | Cor, MTP, CNi, Nic, PaB | 2 |  | WTNP, TEaP |
| *Ctenogobius sagittula* (Günther, 1862) |  | 8 | SCB, MaT, Cor, MTP, CNi, Nic, PaB, Gua | 2 |  | WTNP, TEaP |
| *Elacatinus puncticulatus* (Ginsburg, 1938) |  | 7 | SCB, Cor, MTP, CNi, Nic, PaB, Gua | 2 |  | WTNP, TEaP |
| *Eleotrica cableae* Ginsburg, 1933 |  | 3 | NGI, EGI, WGI | 1 |  | Gal |
| *Evermannia erici* Bussing, 1983 | 1 |  | Nic | 1 |  | TEaP |
| *Evermannia longipinnis* (Steindachner, 1879) | 2 |  | SCB, Cor | 1 |  | WTNP |
| *Evermannia panamensis* Gilbert & Starks, 1904 |  | 3 | CNi, Nic, PaB | 1 |  | TEaP |
| *Evermannia zosterura* (Jordan & Gilbert, 1882) |  | 4 | Cor, Gua, Nic, PaB | 2 |  | WTNP, TEaP |
| *Evorthodus minutus* Meek & Hildebrand, 1928 |  | 6 | Cor, MTP, CNi, Nic, PaB, Gua | 2 |  | WTNP, TEaP |
| *Gillichthys detrusus* Gilbert & Scofield, 1898 | 1 |  | Cor | 1 |  | WTNP |
| *Gillichthys mirabilis* Cooper, 1864 |  | 4 | NCa, SCB, MaT, Cor | 2 |  | CTNP, WTNP |
| *Gillichthys seta* (Ginsburg, 1938) | 1 |  | Cor | 1 |  | WTNP |
| *Gobioides peruanus* (Steindachner, 1880) |  | 3 | CNi, PaB, Gua | 1 |  | TEaP |
| *Gobionellus daguae* (Eigenmann, 1918) | 1 |  | PaB | 1 |  | TEaP |
| *Gobionellus liolepis* (Meek & Hildebrand, 1928) |  | 4 | CNi, Nic, PaB, Gua | 1 |  | TEaP |
| *Gobionellus microdon* (Gilbert, 1892) |  | 6 | Cor, MTP, CNi, Nic, PaB, Gua | 2 |  | WTNP, TEaP |
| *Gobiosoma aceras* Ginsburg, 1939 | 2 |  | CNi, PaB | 1 |  | TEaP |
| *Gobiosoma chiquita* (Jenkins & Evermann, 1889) | 2 |  | Cor, PaB | 2 |  | WTNP, TEaP |
| *Gobiosoma hildebrandi* (Ginsburg 1939) | 1 |  | PaB | 1 |  | TEaP |
| *Gobiosoma homochroma* (Ginsburg, 1939) | 1 |  | PaB | 1 |  | TEaP |
| *Gobiosoma nudum* (Meek & Hildebrand, 1928) |  | 6 | Cor, CNi, Nic, PaB, Gua, EGI |  | 3 | WTNP, TEaP, Gal |
| *Gobiosoma paradoxum* (Günther, 1861) |  | 7 | NCa, Cor, MTP, CNi, Nic, PaB, Gua |  | 3 | CTNP, WTNP, TEaP |
| *Gobiosoma seminudum* (Günther, 1861) |  | 4 | Cor, CNi, Nic, PaB |  |  | WTNP, TEaP |
| *Gobulus birdsongi* Hoese & Reader, 2001 | 1 |  | PaB | 1 |  | TEaP |
| *Gobulus crescentalis* (Gilbert, 1892) |  | 5 | MaT, Cor, CNi, Nic, PaB | 2 |  | WTNP, TEaP |
| *Gobulus hancocki* Ginsburg, 1938 |  | 5 | Cor, CNi, Nic, CIs, PaB | 2 |  | WTNP, TEaP |
| *Gymneleotris seminuda* (Günther, 1864) |  | 6 | MaT, Cor, MTP, CNi, Nic, PaB | 2 |  | WTNP, TEaP |
| *Ilypnus gilberti* (Eigenmann & Eigenmann, 1889) |  | 4 | NCa, SCB, MaT, Cor | 2 |  | CTNP, WTNP |
| *Ilypnus luculentus* (Ginsburg, 1938) | 2 |  | MaT, Cor | 1 |  | WTNP |
| *Lophogobius cristulatus* Ginsburg, 1939 | 2 |  | MaT, Nic | 2 |  | WTNP, TEaP |
| *Lophogobius cyprinoides* (Pallas, 1770) | 1 |  | PaB | 1 |  | TEaP |
| *Lythrypnus alphigena* Bussing, 1990 | 1 |  | CIs | 1 |  | TEaP |
| *Lythrypnus cobalus* Bussing, 1990 | 2 |  | CIs, PaB | 1 |  | TEaP |
| *Lythrypnus dalli* (Gilbert, 1890) |  | 7 | Cor, Gua, MaT, NCa, PaB, SCB, EGI |  | 4 | CTNP, WTNP, TEaP, Gal |
| *Lythrypnus gilberti* (Heller & Snodgrass, 1903) |  | 4 | CIs, NGI, EGI, WGI | 2 |  | TEaP, Gal |
| *Lythrypnus insularis* Bussing, 1990 | 1 |  | Rev | 1 |  | TEaP |
| *Lythrypnus lavenbergi* Bussing, 1990 | 1 |  | CIs | 1 |  | TEaP |
| *Lythrypnus pulchellus* Ginsburg, 1938 |  | 6 | MaT, Cor, Rev, CNi, Nic, PaB | 2 |  | WTNP, TEaP |
| *Lythrypnus rhizophora* (Heller & Snodgrass, 1903) |  | 6 | Rev, CNi, CIs, NGI, EGI, WGI | 2 |  | TEaP, Gal |
| *Lythrypnus solanensis* Acero, 1981 | 1 |  | PaB | 1 |  | TEaP |
| *Lythrypnus zebra* (Gilbert, 1890) |  | 6 | NCa, SCB, MaT, Cor, MTP, Rev |  | 4 | CTNP, WTNP, TEaP |
| *Microgobius brevispinis* Ginsburg, 1939 |  | 5 | MaT, Cor, CNi, Nic, PaB | 2 |  | WTNP, TEaP |
| *Microgobius crocatus* Birdsong, 1968 |  | 4 | CNi, Nic, PaB, Gua | 1 |  | TEaP |
| *Microgobius curtus* Ginsburg, 1939 |  | 4 | CNi, Nic, PaB, Gua | 1 |  | TEaP |
| *Microgobius cyclolepis* Gilbert, 1890 |  | 4 | MaT, Cor, PaB, Gua | 2 |  | WTNP, TEaP |
| *Microgobius emblematicus* (Jordan & Gilbert, 1882) |  | 5 | MaT, Cor, CNi, Nic, PaB | 2 |  | WTNP, TEaP |
| *Microgobius erectus* Ginsburg, 1938 |  | 5 | MaT, Cor, MTP, CNi, PaB | 2 |  | WTNP, TEaP |
| *Microgobius miraflorensis* Gilbert & Starks, 1904 |  | 5 | Cor, MTP, CNi, PaB, Gua | 2 |  | WTNP, TEaP |
| *Microgobius tabogensis* Meek & Hildebrand, 1928 |  | 6 | MaT, Cor, CNi, Nic, PaB, Gua | 2 |  | WTNP, TEaP |
| *Microgobius urraca* Tornabene, van Tassell & Robertson 2012 | 1 |  | Nic | 1 |  | TEaP |
| *Parrella fusca* Ginsburg, 1939 | 1 |  | PaB | 1 |  | TEaP |
| *Parrella ginsburgi* Wade, 1946 |  | 3 | Cor, Nic, PaB | 2 |  | WTNP, TEaP |
| *Parrella lucretiae* (Eigenmann & Eigenmann, 1888) | 2 |  | CNi, PaB | 1 |  | TEaP |
| *Parrella maxillaris* Ginsburg, 1938 |  | 3 | Cor, PaB, Gua | 2 |  | WTNP, TEaP |
| *Quietula guaymasiae* (Jenkins & Evermann, 1889) | 1 |  | Cor | 1 |  | WTNP |
| *Quietula y-cauda* (Jenkins & Evermann, 1889) |  | 4 | NCa, SCB, MaT, Cor | 1 |  | WTNP |
| *Tigrigobius digueti* (Pellegrin, 1901) | 2 |  | MTP, Cor | 2 |  | WTNP, TEaP |
| *Tigrigobius inornatus* Bussing, 1990 |  | 4 | Cni, Nic, PaB, EGI | 2 |  | TEaP, Gal |
| *Tigrigobius janssi* Bussing, 1981 |  | 3 | CNi, Nic, PaB | 1 |  | TEaP |
| *Tigrigobius limbaughi* Hoese & Reader, 2001 | 1 |  | Cor | 1 |  | WTNP |
| *Tigrigobius nesiotes* Bussing, 1990 |  | 3 | CIs, NGI, EGI | 2 |  | TEaP, Gal |
