# Supplementary material for: Gamma-diversity partitioning of gobiid fishes (Teleostei: Gobiidae) ensemble along of Eastern Tropical Pacific: Biological inventory, latitudinal variation and species turnover
Source: PLoS One. 2018 Aug 31;13(8):e0202863. doi: 10.1371/journal.pone.0202863 (PMC6118385; doi:10.1371/journal.pone.0202863)
Supplement: S4 Table — (DOCX) [file pone.0202863.s008.docx]

**S4 Table.** Beta diversity partitioning outputs of species turnover and nestedness components among biogeographic provinces.

|  | **Pairwise comparisons** | **Turnover** | **Nestedness** |
| --- | --- | --- | --- |
| **Global test** |  | 0.527 | 0.322 |
|  |  |  |  |
| **Between provinces** | CTNP-WTNP | 0.000 | 0.882 |
|  | WTNP-TEaP | 0.406 | 0.141 |
|  | TEaP-Gal | 0.333 | 0.557 |
